# Antibacterial approaches in tissue engineering using metal ions and nanoparticles: From mechanisms to applications

**DOI:** 10.1016/j.bioactmat.2021.04.033

**Published:** 2021-05-08

**Authors:** Maria Godoy-Gallardo, Ulrich Eckhard, Luis M. Delgado, Yolanda J.D. de Roo Puente, Mireia Hoyos-Nogués, F. Javier Gil, Roman A. Perez

**Affiliations:** a Bioengineering Institute of Technology, Universitat Internacional de Catalunya, Carrer de Josep Trueta, 08195, del Vallès, Sant Cugat, Barcelona, Spain; b Proteolysis Lab, Department of Structural Biology, Molecular Biology Institute of Barcelona, CSIC, Barcelona Science Park, Baldiri Reixac 15-21, 08028, Barcelona, Spain

**Keywords:** Metal ions, Metal nanoparticles, Mechanism of action, Antibacterial activity, Tissue engineering, Biomaterials applications

## Abstract

Bacterial infection of implanted scaffolds may have fatal consequences and, in combination with the emergence of multidrug bacterial resistance, the development of advanced antibacterial biomaterials and constructs is of great interest. Since decades ago, metals and their ions had been used to minimize bacterial infection risk and, more recently, metal-based nanomaterials, with improved antimicrobial properties, have been advocated as a novel and tunable alternative. A comprehensive review is provided on how metal ions and ion nanoparticles have the potential to decrease or eliminate unwanted bacteria. Antibacterial mechanisms such as oxidative stress induction, ion release and disruption of biomolecules are currently well accepted. However, the exact antimicrobial mechanisms of the discussed metal compounds remain poorly understood. The combination of different metal ions and surface decorations of nanoparticles will lead to synergistic effects and improved microbial killing, and allow to mitigate potential side effects to the host. Starting with a general overview of antibacterial mechanisms, we subsequently focus on specific metal ions such as silver, zinc, copper, iron and gold, and outline their distinct modes of action. Finally, we discuss the use of these metal ions and nanoparticles in tissue engineering to prevent implant failure.

## Highlights

• Distinct mechanisms concertedly contribute to the antibacterial effect of metal ions and nanoparticles in tissue engineering.
• Metal-mediated antibacterial mechanisms include membrane disruption, ROS generation, and protein/DNA damage.
• As different metals/nanoparticles prompt different antibacterial mechanisms, biomaterials may benefit from combinatorial use.
• Application specific antibacterial biomaterials with controlled metal release rates are achievable by combining techniques.

## Graphical abstract

**Figure:**
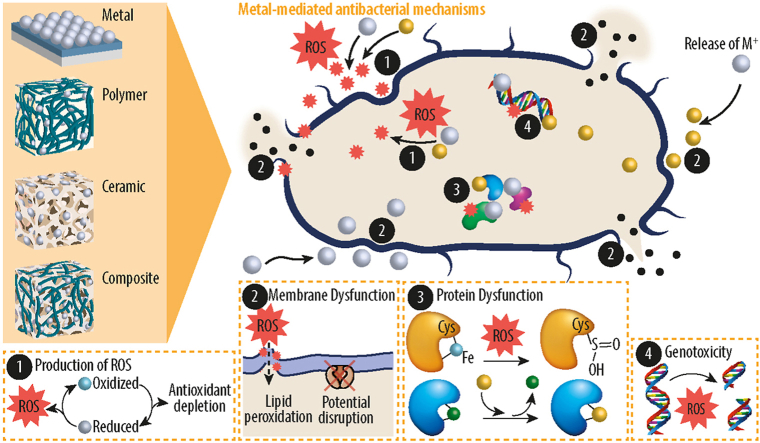

## 1 Introduction

Human tissues have a complex hierarchical structure that may endure trauma, cancer or some degenerative diseases during human lifetime and hence identifying new strategies to regenerate and repair the impacted tissue is of paramount interest. A variety of constructs, mainly biomaterials and tissue engineering scaffolds, are being developed and optimized. Nevertheless, the introduction of external devices or materials into the human body is strongly connected with an increased possibility of a bacterial infection. For example, even the presence of a very limited number of bacteria, such as in operating theatres, may have fatal consequences. These bacterial infections can appear months to years after surgical intervention and can cause implant failure and patient suffering [1]. Thus, advancing biomaterials and constructs that are able to mitigate such adverse bacterial infections are of great interest, and thereupon, it is of outmost importance to understand the underlying mechanisms and antibacterial properties of the various metal ions and metal nanoparticles used, which will be the main focus of this review.

Infectious diseases caused by microbes are an important health and economical issue. For example, the cost for treatment of implant-associated osteomyelitis in the US is expected to exceed $1.62 billion by 2020 [2]. Due to the continuous emergence of bacterial resistance, an increasing number of research is focusing on the development of novel antimicrobial agents. Antibiotics have three predominant bacterial targets: cell wall synthesis, DNA replication, and the protein translational machinery [3]. However bacteria may develop resistance against all these targets. Resistance mechanisms include the expression of enzymes that are able to degrade, modify or inactivate the respective antibiotics (e.g. β-lactamases), modification of the antibiotic's target (e.g. by amino acid mutation or post-translational modification), changing the cell composition and modification or alteration of efflux pumps [3,4].

With the steadily increasing impact of antibiotic resistant bacterial strains, effective and long-term antibacterial materials are desperately needed. Metals have been used throughout the centuries, and have been extensively studied for their antimicrobial properties. Importantly, a number of metals are essential to achieve cellular functions and are thus indispensable for the biochemistry and metabolism of all living entities. Essential metal ions (M^n+^) such as copper (Cu), manganese (Mn), iron (Fe) and Zinc (Zn) are important in the structure of the cell membrane and DNA, and they frequently participate in key cellular processes such as electron transfer and catalysis [5,6]. However, when these essential metals are in excess, their effects can be lethal to cells [7]. On the other hand, non-essential metals such as silver (Ag) or mercury (Hg), are toxic even at low concentrations. Comparable to antibiotics, the effect of metals is distinguishable between bacterial and mammalian targets due to deviating metal transport systems and metalloproteins [5]. This allows the use of metal-based nanomaterials as long-term antimicrobial agents with no or very little detrimental effects on the host.

Several studies have focused on the applications of metal ions and on the synthesis of metal nanoparticles (M-NPs) with potent antimicrobial properties [[8], [9], [10], [11]]. It is suggested that M^n+^ and M-NPs hold great potential to decrease or even eliminate antibiotic resistant bacteria. Reported modes of action include: disruption of the cellular membrane and of protein complexes, and degradation of cellular key components such as DNA and proteins [[12], [13], [14], [15]]. Common materials used include Ag, gold (Au), Cu, Zn and their corresponding oxides. However, there is still a pressing need to further characterize and delineate their mechanism of action, as they are still ill-defined due to the different parameters used in the research performed so far. Thus, it is important to mention that a fully conclusive comparison and interpretation among all the data is currently inaccessible. However, it is evident that several antimicrobial routes are employed simultaneously, and due to the variety of mechanisms that jointly target the microorganism, the development of antibiotic resistance seems highly unlikely.

The risk of a bacterial infection represents an important constraint during the implantation of biomaterials. Importantly, infections at implant or device sites are frequently difficult to treat due to their deep tissue localization and the microorganism involved. Moreover, one of the major clinical complications associated with implants and devices is actually attributed to biomaterial-associated infections (BAIs), which can compromise the function of the implant or device, and lead to increased morbidity and even mortality in patients. In general, biomaterials face two major objectives when implanted into the body: (i) to make a satisfactory integration with the native tissue and restore function, and (ii), to prevent colonization of microbes onto the surface. This is often referred to as “the race for the surface”, which describes the competition between the intended tissue integration and the detrimental attachment of bacteria onto the biomaterial surface [16]. In recent years, various M^n+^ and M-NPs have been incorporated into biomaterials thereby altering their physicochemical properties and providing important antibacterial capabilities to the material. Due to their small size, nanomaterials have a high surface-to-volume ratio, rendering them more effective than their bulk form, and ensuring their functionality even at low concentrations, and thus priming them for the use in metal-doped biomaterials. With the increasing understanding of the underlying antibacterial pathways, together with the cumulating research on the combinatory effect of different metal species, improved biomaterials are on the horizon.

In this review, we summarize and highlight the current state-of-the art of metal ions and nanoparticles, their anti-bacterial properties and their use in biomaterials. Our main focus is the current understanding of the antibacterial mechanism of action by these M^n+^ and M-NPs. We will start by discussing the general principles that guide metal toxicity, and will then describe the various antimicrobial mechanisms employed by metal ions. Finally, we will take a fresh look of their application in the biomaterials field.

## 2 Structure of the bacterial cell wall

**Fig. 1:**
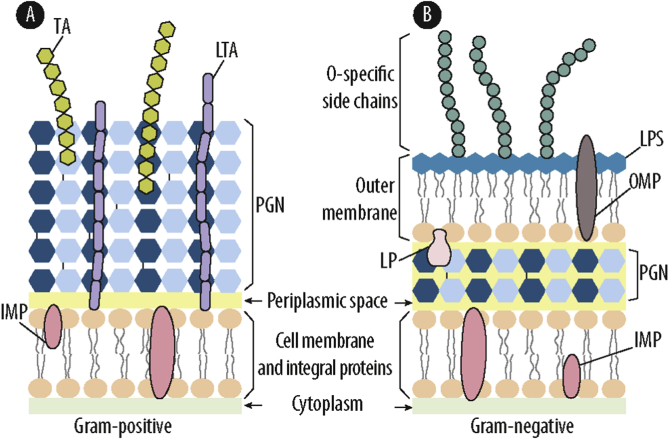
Cell wall schematic of (A) Gram-positive and (B) Gram-negative bacteria. IMP: Integral membrane protein; LP: lipoprotein; LPS: lipopolysaccharide; LTA: lipoteichoic acid; OMP: Outer membrane protein; PGN: Peptidoglycan; TA: wall teichoic acid. **Note**: the schematic is not to scale.

In order to understand the mechanisms of action of metal ions and nanoparticles, it is important to first focus on the bacterial cell structure. Bacteria have developed an elaborated and complex cell wall that protects them from the often-hostile environment while permitting the import and export of selected nutrients and cellular waste products, respectively. The bacterial cell wall is a multi-layered mesh-like structure, predominantly composed of proteins, lipids and carbohydrates. Related to differences in cell wall structure, bacteria are classified based on their Gram-positive or Gram-negative staining [17]. The Gram-positive cell wall consists of a thick peptidoglycan (PGN) layer (20–80 nm) [17] which is densely functionalized with anionic glycopolymers (Fig. 1A). These cell wall contains teichoic acids that are either covalently attached to the PGN or anchored in the bacterial membrane via a surface-associated adhesion amphiphile, namely lipoteichoic acid. The PGN layer is built up by repeating units of the disaccharide *N*-acetyl glucosamine*-N-*acetyl muramic acid, which are cross-linked via pentapeptide side chains, hence forming a thick and robust layer [17]. The Gram-negative cell wall is more complex but contains a thinner PGN layer (7–8 nm) which is placed in-between the cell membrane and the outer membrane (Fig. 1B). The Gram-negative outer membrane consists of negatively charged lipopolysaccharides (LPS), which are exclusive to Gram-negative bacteria. This outer membrane serves as a strong barrier that prevents the entry of hydrophobic substances or macromolecules, but it also contains additional components such as porins that allow the diffusion of selected molecules [15]. Importantly, LPS is a potent and pleiotropic inflammatory stimulus in mammals, typically referred to as endotoxin, and thus it plays a central role in the pathogenicity of Gram-negative bacteria [15]. Due to the low permeability of the outer membrane, Gram-negative bacteria are considered by many researchers in the field to be less sensitive to metal ions and nanoparticles than Gram-positive bacteria which lack this cell envelope structure [8,[18], [19], [20], [21], [22]]. However, the Gram-positive bacterium *Staphylococcus aureus* (*S. aureus*) was reported to be e.g. less susceptible to Cu and Ag nanoparticles than the Gram-negative bacterium *Escherichia coli (E. coli)* [23,24]. Thus, it seems unlikely that the sensitivity of bacterial strains solely depends on the cell wall structure and thus Gram nature, but rather on the individual cell wall composition and thickness.

## 3 General mechanisms of antibacterial metal activity

Metal ions have a wide range of chemical and physical properties that define their mechanism cell toxicity. M^n +^ may associate with distinct targets in the bacterial cell, including enzymes, membranes and DNA molecules. They can exist as many different chemical species depending on factors such as temperature, pH, ionic strength, binding partners and the reduction potential of the local environment. For example, the cytoplasm is a strong reducing environment, especially compared to the periplasm of Gram-negative bacteria. This significantly affects the oxidation state of metals and thus metal speciation. Additionally, metals are typically not freely available inside cells. Instead, a complex network of transporters, metalloregulatory sensors and metallochaperones regulate metal speciation and availability, and ensure the directed transport to e.g. metalloproteins, including metal-dependent enzymes, structural proteins and metal storage proteins which serve as biological reserve [25]. In general, metal speciation strongly influences its bioavailability and reactivity, and thus represents an important physicochemical property for metal toxicity.

Metallic nanoparticles are inorganic particles with sizes ranging from 1 to 100 nm and different shapes (e.g. spherical, triangular, sheets, plates, tubes, cubes and rods) [26]. Importantly, recent research has shown that many factors may influence the antibacterial effects of the M-NPs, such as their size, charge, zeta potential, surface morphology and structure.^28^ The small size of M-NPs is of great advantage for achieving strong antimicrobial activity in the fight against bacteria. For example, smaller M-NPs typically have higher antibacterial activity due to their relatively larger surface to volume ratio, which increases their capability to produce ROS, and which in turn can damage bacterial biomolecules, proteins and lipids. Among M-NPs with identical surface-to-volume ratios, the shape plays an equally important role, where nanotubes and rods are more effective due to the exposition of their planes and thus oxidation of the metals [3,27,28].

**Fig. 2:**
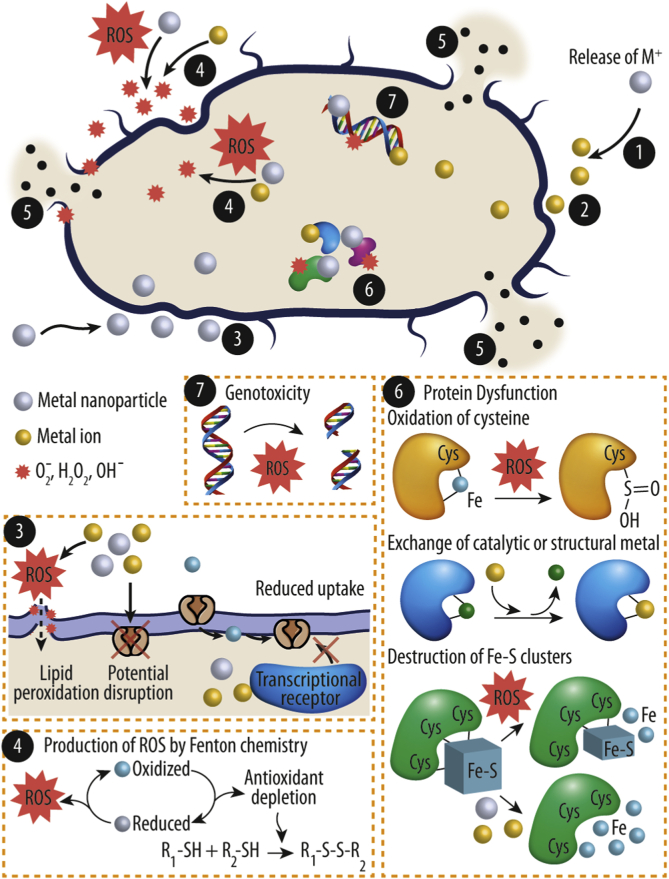
Antibacterial mechanisms of metal ions and nanoparticles. The central modes of action are: (1) release of metal ions from the metal nanoparticles and (2) direct interaction of the metal ions and/or (3) metal nanoparticles with the cell wall through electrostatic interactions, leading to impaired membrane function and impaired nutrient assimilation; (4) formation of extracellular and intracellular reactive oxygen species (ROS), and damage of lipids, proteins and DNA by oxidative stress; (5) high-levels of metal-binding to the cell envelope and high ROS levels can cause damage to the plasma membrane and thus lead to the leakage of the cell content; (6, 7) upon metal uptake, metal nanoparticles and metal ions can directly interfere with both proteins and DNA, impairing their function and disturbing the cellular metabolism in addition to metal-mediated ROS production.

First, the nanoparticles attach to the membrane of the bacteria by electrostatic interactions, van Der Waals forces, receptor-ligand or hydrophobic interactions [29]. After making contact, the M-NPs can cross the bacterial membrane, obstruct metabolic pathways and cause changes in membrane shape and function. Once inside cells, M-NPs can inhibit enzymes, deactivate proteins, induce oxidative stress and modify gene expression levels [29]. Accumulation of metal inside of the microorganism is considered to be a key step in metal toxicity. Alternatively, the applied metal ions can block the uptake of essential ions by impairing the various metal transport mechanisms of the bacterium, or by generating external reactive oxygen species (ROS). It is important to note that the mechanisms of action discussed here are not exclusive, as antibacterial activity is the complex result of multiple and often interconnected mechanism that happen simultaneously. Therefore, it is difficult to delineate their individual contributions in a complex biological system (Fig. 2).

### 3.1 Metal ions and metal nanoparticles induce cell membrane disruption

The first point of interaction between the M^n+^ and M-NPs and bacterial cells is the cell envelope (Fig. 2, point 2 and 3). The cell envelope is not only the main barrier between the cell and the environment, but it also hosts multiple essential functions for cell survival, such as the electron transport chain. This protection is achieved in part by ionogenic components of the bacterial cell that supplies reactive groups that can interact with distinct metals and thus provide the first contact points [30]. The metal interaction with proteins is not only limited to the active site of enzymes, but also membrane proteins often exhibits an anionic net charge, which at circumneutral pH can bind positively charged metals at circumneutral pH.

The number and distinct types of proteins in the bacterial envelope depend on the respective species, its distinct surface composition, developmental stage and structure. For example, an ion channel in species A may be more susceptible to Hg than its counterpart in species B due to the presence of a surface exposed cysteine residue. These maybe even subtle differences are hard to rationalize, but are believed to have a significant impact on the antibacterial effect of the applied compounds, and are thought to explain in part the seemingly contrasting results obtained with the same metal against different bacterial species [11,19,22,31,32].

Lipids are the major component of the bilayer membrane [33]. M^n +^ or M-NPs binding to lipids has an immediate effect on membrane stability and its capability to dynamically reorganize. Phospholipids are the dominant compound of the bacterial membrane and contain reactive phosphoryl groups next to the carboxyl groups of unmodified lipids, both able to interact with metal cations at circumneutral and alkaline pH. Importantly, the composition of the bacterial phospholipids differs within the membranes of different species, and the interaction with the metal mainly depends on the outwards facing, polar headgroup of the lipid, which are specifically susceptible to perturbations. Once the ion binds to the membrane, the membrane dipole potential is reduced, and the hydration of the head group is altered [34]. Thereby, the overall charge of the membrane is altered resulting in local membrane disruption and an increase in permeability as well as ROS formation (Fig. 2, point 4) [32,[35], [36], [37]]. Based on the capacity of membranes to coordinate with M^n+^ and M-NPs, it has been suggested that the bactericidal effect of many metals is related to their binding ability to the cell membrane and to impair its cellular function [12,38].

Additionally, lipid peroxidation is a process where oxidants such as free radicals (e.g products of the Fenton reaction) react with the carbon-carbon double bond of lipids, especially in polyunsaturated fatty acids (Fig. 2, point 4). This process involves the hydrogen abstraction from a carbon and subsequent oxygen insertion, resulting in peroxyl radicals and hydroperoxides. The main effect of lipid peroxidation is the damage of the plasma membrane, decrease of the fluidity and increase of leakage [3,39]. Hong et al. [40] hypothesized that the peroxidation of the membrane phospholipids is one key mechanism of the copper-mediated antibacterial effect.

High levels of ions or nanoparticles can lead to local disruption of the cell barrier, leading to the release of intracellular cell content (Fig. 2, point 5). In an attempt to compensate for the apparent water loss, bacteria increase proton efflux and upregulate electron transport. However, these actions require a high level of ions which in turn generate additional damage to the transmembrane system [41]. The increasing imbalance of ions and instability of the membrane translates in an impaired respiration, thus leading to the disruption of the cellular energy transduction system and ultimately to cell death [42].

### 3.2 Intracellular protein and DNA disruption

Recent studies have shown that intracellular proteins represent likewise prominent targets of metal toxicity due to the many amino acid-mediated binding sites, mainly consisting of reduced thiols from cysteine side chains, carboxy groups of aspartates and glutamates, and the highly-reactive primary amines of lysine side chains [5,12] (Fig. 2, point 6). Upon binding, metal ions catalyze the oxidation of the susceptible amino acids, impairing protein function, reducing protein stability and marking the protein for degradation [5,12]. Furthermore, metal cofactors are required for the proper folding and biological function of many proteins. Additionally, metal ions are highly regulated at homeostasis, to prevent mis-metallation and normal cell function without the formation of ROS [43].

It has been shown that certain metal ions (e.g. gallium ions (Ga)) inhibits bacterial growth or kill the bacterial by a so-called “Trojan horse”-mechanism where the cells take-up another metal ion instead of the essential one due to its similar chemical properties. Once the M^n+^ is inside the cell, it disrupt the metabolic pathways because the cell is not able to reduce it and this irrevocably impairs cell metabolism [44].

Another important mechanism shown for many metals is the indirect damage of bacterial DNA (Fig. 2, point 4). For example, the disruption of Fe homeostasis and the release of free Fe into the cytoplasm increases the amount of intracellular Fenton chemistry, thus increasing ROS levels and accelerating DNA damage by metal-induced oxygen radicals [5].

### 3.3 Generation of reactive oxide species

The generation of ROS is frequently reported in bacterial cells treated with metal ions. These intermediate oxidation-state species are generated by incomplete reduction of oxygen molecules. Consequently, the oxygen-containing radicals are able to exist independently with one or more unpaired electrons. However, the term ROS often includes reactive oxygen containing compounds without unpaired electrons, such as hydrogen peroxide (H_2_O_2_). During homeostasis, ROS levels are tightly controlled and excess is cleansed by the intracellular antioxidant defense system. For example, aerobic respiration inescapably generates a number of reduced species of molecular oxygen such as H_2_O_2_ and superoxide-radical (O_2_^•^‾), that subsequently may interact with intracellular Fe to auto-oxidize [45]. However, when the balance between ROS production and antioxidant defenses is perturbed, the ROS concentration steadily increases and causes severe damage to bacterial proteins, DNA, and lipids, accumulating oxidative stress and leading to cell death [46,47]. Many reports demonstrated that both essential (e.g. Fe (II) and Cu(II)) and non-essential (e.g. chromium (Cr) (VI) and arsenic (As) (III)) ions can increase the intracellular ROS production [12]. Thus, the toxicity of these metals probably derives at least partly from the metal-induced ROS production and the thereby inflicted cellular damage.

The chemistry behind metal-mediated ROS production can be summarized by three major routes (Fig. 2, point 4):

1.- Redox-active metals (i.e. metals that participate in reduction or oxidation reactions by gaining or losing electrons) such as Fe, Cu, Cr and Ni, may play a role in Fenton chemistry, a catalytic process that converts e.g. hydrogen peroxide into a highly toxic hydroxyl free radical [[48], [49], [50]].
2.- Certain metals are capable to disrupt the cellular donor ligand that coordinates iron. In particular, it has been shown that aluminum (Al), Cu and Ag can directly target proteins containing so-called [4Fe–4S] clusters, such as the bacterial-type ferredoxins, and thereby disrupt their electron transfer function in a wide range of metabolic reactions [51]. Additionally, this may result in the uncontrolled release of Fe into the cytoplasm, where it prompts ROS generation.
3.- Metal ions may cause oxidative stress in microorganism by depleting the reservoir of antioxidants. The thiol-mediated reduction of some metal species, for example Fe(III), Cu(II) and Cr(VI), can provoke the generation of ROS through a sulfur radical intermediary. For example, reduced thiols such as glutathione (GSH), represents a key antioxidant in the bacterial cell. However, GSH can be depleted by oxidizing thiophilic metals such as Ag(I), cadmium (Cd)(II), or As(III). Thus, the anti-oxidative defense of the cell is weakened and the vulnerability increases for subsequent metal-mediated ROS [12,52].

### 3.4 Bacterial resistance to metal ions and metallic nanoparticles

Antibiotic resistance occurs when bacteria develop resistance mechanisms that reduce or eliminate the effects of the respective antimicrobials. Infections by antibiotic-resistant bacteria are more difficult to treat, and thus may lead to increased morbidity and mortality. The main antibiotic resistance mechanisms include: (i) active efflux of antibiotics by the overexpression of efflux pumps, (ii) upregulation of alternative metabolic pathways to circumvent those restrained by the antibiotic, (iii) decrease of the bacterial cell wall permeability, thus reducing the income of antibacterial agents to the target site, (iv) expression of enzymes capable of altering or degrading the respective antibiotic, (v) overproduction of the target enzyme to outnumber the antibacterial drug, and (vi) modification of the antibiotic target site. Importantly, horizontal gene transfer of antibiotic-resistance genes through plamids, phages, or the uptake of DNA from the environment, can disseminate antibiotic resistance to other strains and species [3,29,53].

The rise of bacterial resistance requires the development of novel antibacterial agents. In particular, nanomaterials have been promoted as a viable solution to combat antibiotic resistance by eliminating bacteria before they can obtain resistance. Despite the extensive use of M^n+^ and M-NPs, bacterial resistance has been hardly described in the literature. This can be explained by the multiple antibacterial mechanism that are triggered compared to one sole mechanism by a typical drug. Moreover, the small size of the M^n+^ and M-NPs allows them to inflict both extracellular and intracellular damage, and as metal ions and nanoparticles are highly stable, they can target other bacterial cells once they are released from already killed bacteria [54].

Even though bacterial resistance mechanism against metal ions and metallic nanoparticles have not been studied in great depth so far, several potential ways have been discussed in the literature. For example, against particles bigger than 10 nm, it has been shown that bacteria can develop mechanisms via their extracellular matrix, thereby provoking the agglomeration and thus inactivation of the NPs [4,55]. Panácek et al. [56] described the agglomeration of 20 nm Ag-NPs due to the overexpression of the self-polymerizing flagellin protein, and Faghihzadeh et al. [57] detailed the production of extracellular polymeric substances (EPS) that can alter the size and zeta potential of NPs, similarily leading to their agglomeration. Likewise, Siemer et al. [58] studied the interaction of the NPs with biomolecules of the pathophysiological environment, and they could show the formation of a so-called corona on the NPs, which restricted their interaction with the bacteria.

Another reported response mechanism involves the mutational modification or reduction in expression levels of proteins such as porins that are involved in the uptake of silver ions and nanoparticles smaller than 10 nm [4]. For example, Hachicho et al. [59] found in *Pseudomonas putida*, that an adjustment in the unsaturated fatty acids lead over time to a reduced permeability of membrane and thus a lowered uptake of Ag-NPs and ions. Moreover, upregulation of efflux pumps can lead to an improved removal of metal ions released from internalized M-NPs [4]. Similarly, bacteria can upregulate intra- and extracellular metal sequestration and bio-precipitation, increase expression of enzymes for detoxification, or alter cell morphology, to overcome detrimental levels of metal ions [60]. Envelope stress response represents another described mechanism, which involves the modulation of the electrical charge of the bacterial envelope, e.g. by incorporating d-alanine or lipid A, and thereby reducing the negative net charge, or increasing the positive charge of the membrane, respectively [4],

Importantly, when the metal-induced ROS concentration in the bacterial cell is not lethal, an adaptive defence process called hormesis can be triggered. These include short- and long-term adaptations, such as the stimulation of ROS scavenger enzymes which allow the bacteria to maintain their redox balance for additional minutes, and the general upregulation of antioxidant mechanisms, respectively. Simultaneously, DNA repair mechanisms are typically activated [4]. Nevertheless, there exists an increased probability of spontaneous mutations and genome plasticity due to the ROS inflicted oxidative stress, which can at times result in beneficial mutations and thus in the resistance to M-NPs and M^n+^ [4,61,62].

## 4 Antibacterial activity of silver

Silver ions (Ag^+^) are well known to be toxic for bacteria, viruses, fungi and some other organisms [63,64] while they show low or negligible toxicity in humans [65]. The increased attention to silver-based nanoparticles (Ag-NPs) in many scientific areas and for a broad range of applications is due to their beneficial properties. For example, their size-, shape, and surface can be controlled during synthesis, and their biochemical functionality tailored to the respective application [66]. Thus, especially Ag-NPs have been investigated extensively as a powerful nanoweapon for the destruction of bacteria. Silver nanoparticles possess additionally to their own antimicrobial activity also the properties of ionic silver as the latter can be generated by the spontaneous release of Ag^+^ from the nanoparticle surface. Choi et al. [67] proposed the release of Ag^+^ from nanoparticles after interaction with oxygen. In particular, the authors showed that their silver nanoparticles released approx. 2.2% of its silver content into solution after one week of oxygen exposure. Later, Asharani et al. [68] proposed that *in vivo*, the release of Ag^+^ is triggered by the interaction of Ag-NPs with H_2_O_2_.

Despite extensive research on both silver ions and silver nanoparticles, the exact mechanism of antimicrobial action is still elusive. However, the most probable mechanism of silver compounds may include (i) extensive disruption of cellular functions due to direct damage of the cell membrane or (ii) intracellular biomolecules and (iii) the induction of oxidative stress by metal-mediated ROS production, culminating in the formation of free radicals and extensive cellular damage [69,70].

### 4.1 Cell membrane disruption

**Fig. 3:**
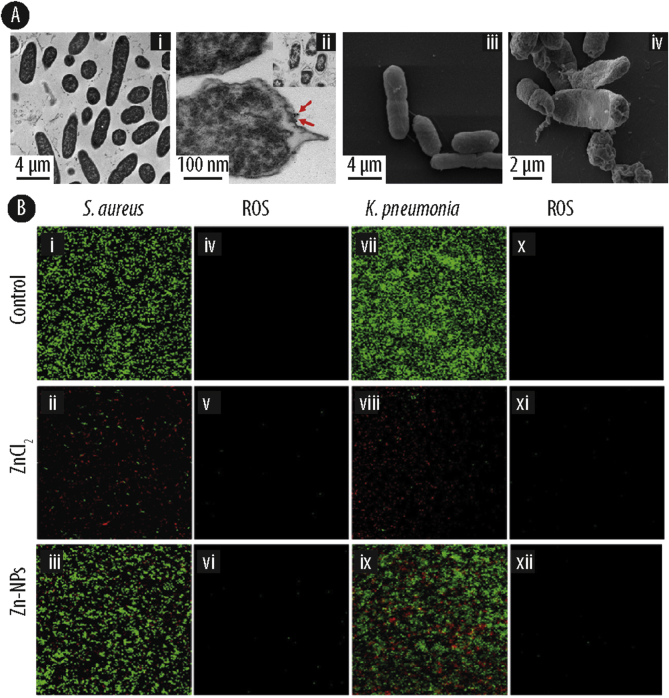
**(A)** Transmission electron microscopy (TEM) (i-ii) and scanning electron microscopy (SEM) (iii-iv) analysis of *Escherichia coli (E. coli)* upon treatment with silver nanoparticles (Ag-NPs) (i and iii: controls; ii and iv: treated samples). **(B)** Dual immunofluorescence and reactive oxygen species (ROS) staining images of *Staphylococcus aureus* (*S. aureus)* (i-vi) and *Klebsiella pneumoniae* (*K. pneumoniae)* (vii-xii) treated with zinc nanoparticles and zinc chloride (0.35 mM) under dark conditions. Reprinted with the permission from Elseiver [78,81].

The interaction of silver species and the bacterial cell begins with the attachment of the silver ions and silver nanoparticles to the cell wall and membrane due to the electrostatic attraction between the negatively charged bacterial surface and the positively charged silver compounds [71]. This charge interplay between the bacterial cell and the silver species can then induce a change in the zeta potential of the cell surface [72], provoking an increase in cell membrane permeability, membrane depolarization and a decreased respiratory potential. Finally, a comprehensive disturbance of membrane integrity leads to irreversible cell damage and consequently to cell death [73]. In the case of Ag-NPs, even only a brief contact with the bacterial cell wall leads to dense pits and strong peripheral damage on the bacterial surface [74]. These changes in the structural properties of the cell wall have been well documented by several microscopy techniques [[75], [76], [77], [78]]. For example, Alsammarraie et al. [78] investigated the changes in bacterial cell morphology upon treatment with Ag-NPs by transmission electron microscopy (TEM) and electron scanning microscopy (SEM) (Fig. 3A). While the untreated cells had smooth and regular cell walls with a homogeneous cytoplasmic cell content, cells exposed to Ag-NPs exhibited huge disruption features and irregular pits in their cell wall in addition to the apparent loss of cytoplasmic material. These results were consistent with previous studies on the effect of Ag-NPs on bacterial cells [79,80].

Depending on the composition of the bacterial cell wall and membrane, distinct pathways for silver ions and nanoparticles have been suggested and described. Most Ag^+^ and Ag-NPs show lower antibacterial activity against Gram-positive than Gram-negative bacteria, probably due to the thicker PGN layer [80,82]. In the case of Gram-negative bacteria, the negatively charged LPS layer is reported to promote the capture and attachment of silver species [83]. Bovenkamp et al. [19] studied the physicochemical form of silver by X-ray absorption near-edge structure (XANES) spectroscopy in both Gram-positive *S. aureus* and Gram-negative *E. coli* after silver ion exposure. The authors suggest that a substantial amount of the therapeutic silver ions and silver nanoparticles bond to the PGN and LPS layer of the bacterial cell wall, and a small portion actually entered the cell, generating intracellular damage. Intriguingly, the thick PGN layer of Gram-positive bacteria represented a better barrier than the LPS of Gram-negative bacteria against Ag^+^ and Ag-NPs.

Both silver nanoparticles and ionic silver may interact with proteins associated to the bacterial cell wall and membrane and thereby form detrimental complexes that alter its physicochemical properties. Silver quickly reacts with the sulfhydryl groups on the bacterial cell membrane by exchanging the terminal hydrogen atom, generating a stable S–Ag bond and thereby fully blocking the respiratory chain, electron transfer, protein secretion and lipid biosynthesis [35,[84], [85], [86]]. Bondarenko et al. [35] demonstrated that the bacterial membrane of both *E. coli* and *Pseudomonas aeruginosa (P. aeruginosa)* is the main target of the Ag-NPs, mostly mediated via released Ag^+^ ions.

### 4.2 Intracellular protein and DNA disruption

The initial extent of damage provoked by the ionic silver and/or silver nanoparticles to the bacterial envelope is crucial for the subsequent cellular entry, where the nanoparticles and silver ions can inflict additional damage on vital cellular functions [87]. One proposed mechanism of the antimicrobial activity of silver nanoparticles is the release of silver ions from its surface, which can diffuse free and bind to biomolecules, thereby altering the function of proteins and nucleic acids [5,15]. It has been shown that binding of Ag-NP to relaxed bacterial DNA leads to the induction of its condensed state, and a total loss of DNA replication [80]. Additionally, silver ions and silver nanoparticles can also provoke DNA denaturation and degradation. Importantly, energy dispersive X-ray analysis (EDX) demonstrated the involvement of sulfur in the underlying biochemical mechanism, which hints the association of silver ions with thiol groups of nucleoid-associated proteins. These result suggest the alteration of chromatin-regulating proteins, thus impairing mRNA transcription and DNA replication [80,82].

Yan et al. [88] studied the effect of Ag-NPs and Ag ^+^ ions on *P. aeruginosa* through a comprehensive analysis of silver-regulated and silver-binding proteins by a combination of proteomic and bioinformatic techniques. The authors identified in total 59 silver-regulated proteins (e.g. adenosine triphosphate (ATP) synthase subunit C, porin D or cytochrome C oxidase) and 5 silver-binding proteins (chaperonin, elongation factor Tu, flagellin, electron-transfer flavoprotein subunit alpha and uncharacterized protein PA3309). Many of these proteins are involved in metal transport, flagellum assembly, pore formation, and membrane stabilization. Based on these findings, two main pathways for the antimicrobial activity of silver were suggested by the authors: (1) the interference with the cell membrane and its thereby impaired functionality (described in this section) and (2) the generation of both extracellular and intracellular ROS and the associated oxidative damage (described in section 4.3). Moreover, some silver-binding proteins were observed for both silver ions and nanoparticles, indicating that Ag-NPs indeed act at least partly via the release of silver ions. Another study [89] also tested the effect of Ag-NP on *E. coli*, and reported the various proteome interactions and changes upon exposure to the silver compound. The authors noted that 65% of the Ag-NPs interacting proteins were enzymes, such as tryptophanase and alcohol dehydrogenase, while the remaining 35% corresponded mainly to membrane porins, chaperones, and peptide-binding proteins.

Another prominent target of silver appears to be the bacterial ribosome. Yamanaka et al. [90] used energy filtered TEM (EFTEM) to verify the infiltration of *E. coli* by the applied silver ions, and used two-dimensional gel electrophoresis (2-DE) in combination with MALDI-TOF MS to study its effect. Authors found that various ribosomal subunit proteins such as the S2 protein were strongly affected by silver ions. Results suggested that silver-induced decrease of S2 protein leads to impaired ribosomes and thus protein synthesis. Consequently, the production of key cellular enzymes such as the citric acid cycle enzyme succinyl-CoA synthetase and fructose-bisphosphate aldolase of the glycolysis and gluconeogenesis pathways were suppressed. Thereby, the cells get increasingly depleted in ATP, leading to cell starvation and ultimately to cell death.

### 4.3 Generation of reactive oxide species

An important aspect of metal-induced antibacterial activity is the generation of reactive oxygen species by silver ions and silver nanoparticles [91]. ROS generation and oxidative stress are the most widely accepted mechanism for the toxicity of Ag-based compounds [92]. Several key studies have found that after reaching the bacterial cell membrane, free radicals generated on the surface of the Ag-NPs would interact with the membrane proteins and oxidize the unsaturated fatty acids. This strong oxidative damage consequently interferes with the fluidity and stability of the membrane [93,94]. Moreover, even cell membrane rupture caused by surface-associated ROS production has been reported [95].

Long et al. [96] studied *E. coli* cell morphology upon treatment with Ag-NPs in the presence and absence of 1 mM GSH. The authors showed that bacteria provided with extra GSH were smooth and intact and closely resembled the non-treated control samples, suggesting that the cell structure collapse in Ag-NPs treated cells were indeed caused by oxidative stress.

Furthermore, Zhang et al. [9] investigated the relationship between distinct Ag-NPs and ROS. For that, the Gram-negative bacteria *Azotobacter vinelandii* (*A. vinelandii*) and *Nitrosomonas europaea* (*N. europaea*) were exposed to Ag-NPs and ROS production was measured by a fluorescence assay based on the oxidation-dependent transformation of non-fluorescent 2′,7′-dichlorofluorescein diacetate (H_2_DCFDA) into the highly fluorescent 2′,7′-dichloro-fluorescein (DCF). The results showed that the generation of ROS strongly correlated with particle size and Ag-NP concentration. At 10 mgL^−1^ of 10 nm Ag-NPs, the authors observed a cell death rate of 20.23% and 1.87% for *A. vinelandii* and *N. europaea*, respectively, while the necrosis rate was 15.20% and 42.20%. So even though in both cases approx. 40% of the cells were killed, different molecular mechanisms were triggered. This could be partly explained by the lower sensitivity of *A. vinelandii* towards the Ag-NPs, which showed a 3-fold lower minimum inhibitory concentration (MIC) compared to *N. europea* (4 mgL^−1^ vs. 12 mgL^−1^). Additionally, TEM images showed a significantly damaged cell morphology and documented a leakage of intracellular content for both bacteria. Thus, these data support the notion that ROS and oxidative stress provokes a wide variety of cellular responses including cell membrane breakage and the induction of cell death.

Additionally, the silver-generated ROS can cause dysfunction of the electron transport chain and proton motive force due to the inactivation of membrane bound enzymes. Importantly, damaging the electron transport chain has an ultimate impact on ATP synthesis, thereby affecting many vital cell functions [[97], [98], [99]].

Apart from disrupting the cell membrane and impairing respiratory enzymes, increased levels of ROS have been observed to damage various cellular components such as DNA, proteins and other biomolecules [100]. Detected DNA damages involve deletions, insertions, point mutations, single-strand breaks, double-strand breaks, fragmentation, and adduct formation. Also altered interaction with DNA-binding proteins are reported [27,101]. After DNA damage, the cell initiates repair by various dedicated cellular mechanisms, as extensive DNA damage inevitably leads to cell death. Tian et al. [102] studied the alteration of the redox balance and the induction of DNA damage in *E. coli*, *S. aureus*, *Lactobacillus bulgaricus (L. bulgaricus)* as well as *Lactobacillus casei (L. casei).* In particular, the authors studied the dissolution of the Ag-NPs and the mediated ^•^OH production that causes oxidative damage to biomolecules. To determine the amount of ^•^OH induced by Ag-NPs, electron spin resonance (ESR) was used. The results showed that the formation of ^•^OH radicals strongly depended on the pH. *Lactobacilli* strains produce and secrete lactic acid which subsequently decreases the pH of the medium, thus favoring the extracellular silver-mediated ^•^OH production. Furthermore, the authors incubated Ag-NPs and AgNO_3_ with GSH, and the products of GSH oxidation (glutathionyl radicals (•SG)) were monitored using ESR. The results indicated that Ag-NPs had a limited effectiveness on GSH oxidation while AgNO_3_ caused a strong oxidation of GSH. Additionally, the authors suggested based on their study that Ag^+^ ions may disrupt the intracellular Fe–S clusters, causing the intracellular release of Fe^2+^ which then further enhances the formation of ^•^OH via Fenton reaction.

## 5 Antibacterial activity of zinc

Zinc ions (Zn^2+^) are involved in the regulation of cell proliferation, differentiation or conservation of the membrane structure of bacterial cells [103]. Moreover, they take part as co-factors in many important metabolic pathways such as the synthesis and degradation of sugars, lipids and proteins [104]. At low concentrations, the beneficial effects of zinc are dominant, while high concentrations actually inhibit bacterial growth. For example, an excess of Zn^2+^ may compete with other metals and provoke a metal mismatch in various metal-binding proteins [105], resulting in protein malfunction, enzymatic inactivation or protein denaturation, thus throwing the bacterial cell off balance [106].

Several studies suggest that Zn^2+^ ions released to the medium from the ZnO-NPs are the main contributor of the antibacterial activity of the respective nanoparticles [107,108]. Pasquet et al. [109] proposed two main determinants for Zn^2+^ release: (i) the physicochemical properties of the nanoparticles such as porosity, concentration, particle size and morphology, and (ii) the chemistry of the media used, e.g. pH, UV illumination, exposure time, and the presence of other chemical elements and compounds. Along this line, Joe et al. [81] proposed that the teichoic acid on the PGN layer of Gram-positive and lipoteichoic acid on the outer membrane of Gram-negative bacteria facilitate the dissolution of ZnO-NPs and thereby the release of Zn^2+^ via formation of ionic salts. Ahmed et al. [22] studied the release of Zn^2+^ as a result of the interaction of bacterial metabolites with ZnO-NPs adsorbed to the bacterial surface. After 24 h of incubation with different bacterial strains, the soluble zinc in the bacterial supernatant was different for each studied strain (79.28 ± 12.15 μg mL^−1^ for *E. coli*; 84.14 ± 8.4 μg mL^−1^ for *P. aeruginosa*; 74.56 ± 3.2 μg mL^−1^ for *S. aureus*; and 94.15 ± 6.2 μg mL^−1^ for *K. pneumonia*), while different amounts were observed in the acid digested bacterial cell pellet (598.4 ± 24.5, 612.3 ± 14.6, 590.6 ± 17.5, and 635.2 ± 21.2 μg mL^−1^, respectively).

Despite intensive studies, the toxicity mechanism mediated by zinc oxide nanoparticles (ZnO-NPs) is still disputed. While several researchers believe that their toxicity is mainly linked to the release of Zn^2+^ ions [110,111], others attribute the antimicrobial effect directly to the nanoparticles itself [112,113]. Like other NPs, ZnO-NPs are thought to kill bacteria by damaging the cell membrane as well as the metal-mediated oxidative stress. The different mechanisms proposed in the literature are listed as: (1) loss of cellular integrity by direct contact of ZnO-NPs with the cell wall and/or cell membrane and (2) release of Zn^2+^ upon ZnO-NPs dissolution and following by intracellular ROS production subsequent modification and damage of biomolecules [81,114,115].

### 5.1 Cell membrane disruption

There is strong evidence that one of the main mechanism underlying the antibacterial effect of ZnO-NPs is based on the binding of the NPs to the bacterial surface and its accumulation in the cytoplasm. Indeed, contact between ZnO-NPs and the cell wall appears to be sufficient to provoke bacterial toxicity.

The impact of the Gram-nature on zinc toxicity was studied in great detail by Tayel and co-workers [20]. The authors found that Gram-positive bacteria are more susceptible to ZnO than Gram-negative bacteria, confirming the MIC of ZnO-NPs obtained by Reddy et al. [21] for *S. aureus* (1 mg mL^−1^) and *E. coli* (3.4 mg mL^−1^). Similarly, Pati and co-workers [116] and Agua et al. [117] demonstrated the higher susceptibility of Gram-positive bacteria and the reduced diffusion of ZnO-NPs through the hydrophobic cell wall of Gram-negative bacteria (*Mycobacterium bovis-BCG*), likely explaining their higher resistance. However, Ahmed et al. [22] demonstrated a higher toxicity of ZnO-NPs on the Gram-negative bacteria *E. coli*, *Klebsiella pneumoniae* (*K. pneumoniae)* and *P. aeruginosa* compared to the Gram-positive bacterium *S. aureus*. Results indicate that the strength of Zn compounds may depend on the respective ZnO-NP and the sensitivity of the individual microorganism, and does not solely rely on the Gram-nature of the organism.

The interaction of ZnO-NPs and Zn^2+^ with the cell surface is attributed to the positive charge of the zinc compound and the overall negative-charge of the bacterial membrane [53,118]. Such reverse charges enhance the attraction by creating electrostatic forces, resulting in a strong ionic bond between zinc and the bacterial surface. Additionally, once Zn^2+^ is bound to the bacterial membrane, it may lead to increased membrane permeability, resulting in a higher probability of ZnO-NPs to enter the bacterial cell which triggers additional cellular responses. ZnO-NPs can equally create pores in the bacterial surface, thus breaking cell membrane integrity and causing a leakage of cytoplasmic material into the exterior, provoking the induction of cell death. Brayner et al. [36] studied the interaction between *E. coli* and ZnO-NPs and observed an induced disorganization of the cell membrane, the internalization of NPs into the bacterium, and an overall reduction of bacterial growth. Similar results were reported by Lallo da Silva et al. [10] with *S. aureus.* After exposure to ZnO-NPs, the bacterial cells exhibited membrane holes, and the same effect was reported by Ahmed et al. [22] who observed extensive membrane disorganization after ZnO-NPs treatment in *E. coli*, *K. pneumoniae*, *P. aeruginosa*, and *S. aureus*.

### 5.2 Photocatalytic bactericidal activity – generation of reactive oxide species

Several studies suggest that similar to silver ions, the main mechanism contributing to antimicrobial activity is the oxidative stress caused by metal-induced ROS production [[119], [120], [121]]. Importantly, as a semiconductor, the electronic structure of ZnO is composed by a conduction band (CB) and a valence band (VB). When ZnO is exposed to UV or visible light with a greater energy than the bandgap (*i.e.* higher than 3.3 eV), electrons can transition from CB to VB [122]. Thereby, positive holes (h^+^) are formed in the VB while free electrons (e^−^) are in the CB. Holes then act as a strong oxidant that can dissociate water molecules into H^+^ and OH^−^. Similarly, electrons act as strong inhibitors that react with dissolved oxygen molecules and produce superoxide radical anions (O_2_^•^). At the same time, these O_2_^•^ interact with H^+^ and form (HO_2_^•^) radicals, which, after interaction with electrons, form hydroxyl peroxide anions (HO_2_^¯^). Finally, these anions interact with hydrogen ions and generate H_2_O_2_ [119,122,123]. Importantly, all these radicals can act as strong oxidizing agents. However, anions cross the negatively charged bacterial cell wall and membrane as they possess the same negative charge, while H_2_O_2_ easily penetrates into the cytoplasm increasing the intracellular ROS levels, causing oxidative stress and subsequent cellular damage by lipid peroxidation, protein denaturation and DNA damage [119,122]. Via these routes, ZnO particles can substantially damage the bacterial cell even without actually entering the cytosol. In this regard, D’Água et al. [117] suggested that H_2_O_2_ could be the main element of the antibacterial activity of ZnO-NPs, especially as they could show that bacteria more sensitive to H_2_O_2_ are also more susceptible to ZnO-NPs. However, Kadiyala et al. [115] found that ROS toxicity played only a minor role in the antibacterial properties of ZnO-NPs against *methicillin-resistant S. aureus*. Instead, they proposed a new mechanism in which ZnO-NPs profoundly impact the anaerobic carbohydrate metabolism and thus bioenergetics, implying a strong biomimetic mode of action of NPs. Whereas they attributed only a minimal impact to zinc ions and zinc-mediated ROS production.

The chemistry behind ROS generation by ZnO is well-defined as several studies reported this mechanism under UV or white light, whilst others also observed it in dark conditions [22,81,114]. Raghupahi et al. [114] reported that the antibacterial activity of nanoparticles via ROS production was only induced after exposure with UV light. However, by testing ZnO-NPs particles against *E. coli*, Hirota and co-workers [124] identified antibacterial properties even under dark conditions, and attributed the activity to superoxide anions generated by the nanoparticle surfaces. Recently, Joe et al. [81] compared the ROS production under dark conditions and UV light. Based on their results (Fig. 3B), the ZnCl_2_-treated bacterial population was lower than the ZNO-NPs treated population, implying that the faster release of Zn^2+^ from ZnCl_2_ inhibited the bacterial proliferation at the initial period of incubation. Therefore, the antimicrobial mechanism of ZnO-NPs under dark conditions may not be attributed to the generation of ROS products but rather originate from dissolved Zn^2+^ ions.

## 6 Antibacterial activity of copper

Copper is an important cofactor for several key enzymes involved in respiratory and photosynthesis processes, such as cytochrome C oxidase and ceruplasmin, and it is involved in different roles depending on its oxidation state. While the reduced Cu^+^ has affinity to thiols and thioether groups, as found e.g. in cysteine and methionine side chains, the oxidized Cu^2+^ favors the coordination by oxygen or nitrogen groups, found in aspartate and glutamate, or the imidazole ring of histidine, respectively. Consequently, copper can exercise many different roles by interacting with various proteins, and thus it plays a key role in many biological processes [125,126]. Similar to other metal ions, the precise mechanism of the antimicrobial activity displayed by copper ions remains unclear, but several lines of probably intertwined pathways are suggested, and it is expected that a sequence of different pathways lead to bacterial cell death, including disruption of the cell membrane, intracellular alteration of biochemical processes and induction of DNA damage [127,128].

### 6.1 Cell membrane disruption

Copper nanoparticles (Cu-NPs) have been demonstrated to have a variety of antibacterial effects on bacteria, including adhesion to bacterial cell walls via electrostatic interaction, impeding the integrity and function of the cell membrane and the associated proteins. Upon intracellular uptake, Cu-NPs induce the denaturation of intracellular proteins and interaction with sulfur-containing biomolecules and compounds. However, it is also well described that copper ions are gradually released from metal surfaces and Cu-NPs, and subsequently absorbed through the cell membrane, thus allowing direct interaction with functional groups of intracellular proteins and nucleic acids [127,129]. Thus, Cu-NP antimicrobial activity is supposed to originate from both the nanoparticles itself and the released copper ions. Similar to the Cu-NPs, the positively charged copper ions are also attracted to the negatively charged cell membranes [128,130].

As for the other metals discussed in this review, different hypotheses exist for the mechanism of copper-induced bacterial killing. For example, it is suggested that binding of copper to the phospholipids may alter the physicochemical properties of the membrane, thereby decreasing e.g. membrane fluidity and/or flexibility. Moreover, this may increase the oxidative stress due to the increase of hydroxyl radicals at the membrane surface, and may disturb the electron transfer chain via direct or indirect interaction with the quinone pool [131]. Calvano et al. [37] demonstrated that the release of copper ions from a metallic surface leads to dramatic membrane damage, for which a complete membrane degradation into lipids could be observed in *E. coli* after treatment with soluble copper salt. These results suggest that the oxidation of membrane lipids is the primary endorser of bacterial killing by copper ions and Cu-NPs, either by membrane disruption and degradation, or upon uptake, by obstruction of cell growth and division.

Zanzen et al. [31] aimed to elucidate the exact effect mechanism of Cu^2+^ and Cu ^+^ solutions in *S. aureus*, *E. coli* and *P. aeruginosa*. In particular, the authors followed the copper speciation by XANES spectroscopy at the Cu K edge. The results revealed different Cu K-XANES spectra for the tested Gram-positive and Gram-negative bacteria, and clearly demonstrated that Cu^+^-S bond formation represents a crucial consequence of the antibacterial activity of copper, similar to what has been seen for silver ions. Moreover, the absorption and differentiated spectra revealed that the less abundant Cu^+^ represents the dominant form that interacts with the bacterial biomolecules, probably explaining the lower antibacterial efficiency of copper compared to silver, as the latter preferentially exists as Ag(I). Finally, the authors confirmed with their study that the main binding partner of Cu^+^ is sulfur while bonds with phosphate groups such as in ATP play an ancillary role or don't even take place.

**Fig. 4:**
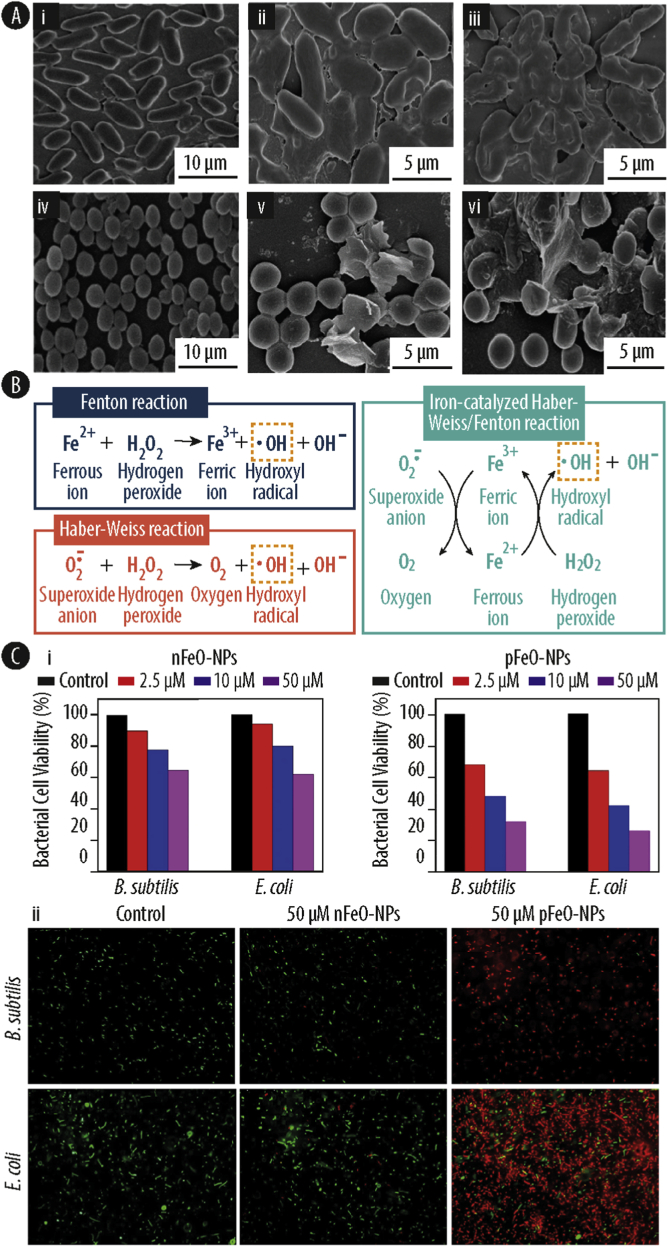
**(A)** Field emission scanning electron microscope (FESEM) images of *Escherichia coli (E. coli)* (i-iii) and *Staphylococcus aureus* (*S. aureus)* (iv-vi) treated with copper at 0h (i and iv), 2h (ii and v) and 24h (iii and vi). **(B)** Fenton and Haber-Weiss reaction for generation of hydroxyl radical. Intracellularly, hydroxyl radicals are primarily produced by iron-catalyzed Haber-Weiss/Fenton reaction. **(C)** (i) Cell viability of *Bacillus subtilis* (*B. subtilis)* and *E. coli* after treatment with negative iron oxide nanoparticles (nFeO-NPs) (left) and positive iron oxide nanoparticles (pFeO-NPs) (right) at different concentrations; (ii) Fluorescence microscopy images of *B. subtilis* and *E. coli* in absence and presence of nFeO-NPs and pFeO-NPs using the LIVE/DEAD BacLight fluorescence kit (green fluorescence: viable cells; red fluorescence: dead cells). Reprinted with the permission from Royal Society of Chemistry [8], CellPress [134], and Nature [135].

Most studies addressing the antibacterial activity of Cu-NPs and copper ions, use both Gram-positive and Gram-negative bacteria to elucidate the impact of their different cell wall structure on metal susceptibility and antibacterial efficiency [8,31,127]. Gram-positive bacteria such as *Bacillus subtilis* (*B. subtilis)* and *S. aureus* have a large amount of amines and carboxyl groups on their cell surface, which exhibits a high affinity to copper ions and copper containing compounds [129,132]. Furthermore, it has been demonstrated for Gram-negative bacteria that the antibacterial activity of copper greatly originates from its redox activity in the periplasmic space [133]. Rauf et al. [8] studied the effect of nanofibers consisting of a copper(II)-based coordination polymer against *E. coli* and *S. Aureus* (Fig. 4A). The authors could demonstrate a significantly higher antibacterial activity and increased membrane damage against *E. coli* compared to *S. aureus*, which they attributed to the thinner cell wall of Gram-negative bacteria.

### 6.2 Intracellular protein and DNA disruption

Traditionally it was assumed that intracellular copper toxicity occurs predominantly through metal-mediated ROS generation. However, recent studies have highlighted intracellular proteins as direct targets of copper toxicity [136]. Copper ions have a high affinity to nitrogen-, oxygen- and sulfur-based donor groups which are all abundantly present in proteins. Given the vast amount of potential binding sites in proteins and considering copper's strong affinity and reactivity, it is highly likely that increased levels of copper ions would lead to impairment of protein function by modification of exposed sulfur groups and by metal displacement in enzymatic and structural metalloproteins [[137], [138], [139]].

Johnson et al. [137] demonstrated that oxidative stress is not the critical mechanism of copper toxicity in S*treptococcus pneumoniae* (*S. pneumoniae)*. Their results showed that copper inhibits by mis-metallation the aerobic nucleotide synthesis pathway, thereby throwing the cells out of balance. While the cell tries to remove the metal from the cytoplasm, the inhibition of the ribonucleoside-diphosphate reductase NrdF decreases cell replication and bacterial growth. This is reinforced as the cell bundles its efforts to up-regulate the transcription machinery of the genes involved in the anaerobic nucleotide synthesis pathway to bring the dNTP pool back to normal. However, other cellular pathways may be impaired in parallel by mis-metallation with copper. Additionally, copper frequently binds to proteins at so-called atypical metal binding sites, which provide only two thiol groups as ligands, and result in a linear biscysteinate coordination [140].

Beyond the mis-metallation and thus inactivation of enzymes, copper toxicity can also be attributed to the disassembly of metal clusters. Iron-sulfur cluster proteins have been shown to be highly vulnerable to copper, especially as they represent key metabolic enzymes in several vital physiological processes and thus are regarded as important targets in copper-mediated antimicrobial activity [141]. Inactivation of iron-sulfur proteins can have a huge impact on physiological functions, spanning from energy metabolism to DNA replication and repair [142]. For example, Djoko et al. [143] established that Cu ions can inhibit the iron-sulfur cluster protein HemN, an enzyme involved in heme biosynthesis. Additionally, Tan et al. [144] described that inhibition of iron-sulfur proteins does not require the presence of oxygen, and that iron-sulfur cluster biogenesis is the primary target of copper-mediated antimicrobial activity in anaerobic cells. Another prominent example is the enzyme isopropylmalate dehydratase, which is involved in the biosynthesis of branched-chain amino acids, which is readily inhibited by copper [141]. Additionally, protein damage also occurs by the disruption of the protein structure. For example, copper can inactivate RNAse A by catalyzing the formation of a non-native disulfide bond in the protein [138]. Similarly, copper may inhibit the reduction of existing disulfide bonds, and thus disturb or stop the maturation of proteins [145].

Little information is available about the interaction of copper with DNA inside the bacterial cell. Although it is known that copper can bind to some copper-sensing transcriptional repressors such as CsoR and RicR [146], the amount of copper that directly interacts with bacterial DNA is not defined. In *E. coli*, it has been demonstrated that DNA is a minor target of copper ions due to a low DNA damage that has been found after extended copper exposure, even in the presence of H_2_O_2_. However, Ananth et al. [147] described that copper ions released from CuO-NPs, can bind to the DNA double helix and disorder DNA strands, eventually affecting both transcription and replication and leading to cell death due to the inflicted DNA damage.

### 6.3 Generation of reactive oxide species

High concentrations of copper are toxic for prokaryotic cells, partly due to its redox properties. Many studies linked the antibacterial activity of copper to its capacity to transition between Cu^+^ and Cu^2+^, which can produce ROS under aerobic conditions. The Fenton chemistry of copper details the decomposition of H_2_O_2_ in ^•^OH, leaving the catalytic metal in its oxidized state. However, as Cu^2+^ is the preferred oxidation state of copper in solution, copper alone is not enough for sustaining the redox reaction. Therefore, a reducing agent (^•^O_2̄_‾, NADPH oxidase from the respiratory chain or intracellular thiols) is needed to return Cu^2+^ to Cu^1+^ to complete the redox cycle and to continue with the ^•^OH production [136]. These free oxygen radicals then may cause lipid peroxidation damage, decreasing membrane fluidity and leading to membrane rupture. Therefore, it is not unlikely that the increased levels of cellular ROS are linked to the damage of the bacterial cell envelope and thus to the antibacterial activity of copper.

Li et al. [148] studied the effect of copper-induced ROS production by testing Ti6Al4V5Cu alloy against *S. aureus*. The authors showed that copper ions were released from the alloy and increased the overall permeability of the plasma membrane, thereby provoking membrane breakage and subsequent leakage of intracellular proteins as well as reducing sugars from the bacterial cytoplasm. The authors also evaluated the ROS concentration of bacteria by the H_2_DCFDA probe, which is oxidized into highly fluorescent DCF upon exposure to intracellular ROS. Their experiments showed a significant increase of fluorescence intensity, pinpointing the ROS formation. This trend became even more evident upon longer exposure times. Additionally, generated free radicals caused lipid peroxidation of the cell membrane, substantially altering its physical properties by decreasing its integrity and fluidity and leading to membrane disruption.

Due to the affinity of copper ions to both the protein backbone and the side chains of several amino acids and the induced higher cellular level of H_2_O_2_, copper-mediated protein oxidation may represent an additional toxicity mechanism. Free radicals can bind to proteins, where the side chains of arginine, proline, lysine and threonine are most susceptible to carbonyl formation [136]. However, it is not clear at this point if protein carbonylation leads to protein damage, or if damaged proteins are more vulnerable to ROS-mediated protein carbonylation [149].

DNA represents one of the main targets of ROS. However, genotoxicity provoked by Cu is controversial. Warnes et al. [150] proposed that the Cu toxicity for *Enterococcus faecalus* and *Enterococcus faecium* involves the direct and indirect action of copper ions and the generation of ROS, culminating in the malfunction of the respiratory chain and DNA repair. However, the authors suggest that the generation of ^•^OH by Fenton chemistry is not the main mechanism of DNA damage, while they propose Cu(II)-induced denaturation of the bacterial DNA as the key causative working. Later, the same authors evidenced the DNA damage of *methicillin-resistant S. aureus* (MRSA) and *methicillin-sensitive S. aureus* (MSSA) by a genomic DNA fragmentation assay and confirmed the role of ROS [48]. However, Mathews et al. [151] suggested that ROS were not the main cause of cell death by copper-based compounds. Instead, the authors concluded that the cell death was primarily provoked by the attack of iron-sulfur clusters.

While Cu ions are known to be capable of ROS formation, the capacity of Cu-NPs to do the same is not yet well understood. In *in vivo* situations and biological systems, a huge variety of substances can bind or be adsorbed to the surface of the Cu-NPs, thus impacting their reactivity. Sulce et al. [152] demonstrated the ability of Cu-NPs to form ROS from H_2_O_2_. However, the authors hypothesized that the formation of ROS was linked to the oxidative decomposition of the Cu-NPs, and actually originated from the thereby formed reactive Cu ions.

## 7 Antibacterial activity of iron

Iron is an essential microelement for bacterial life and involved in many biological pathways such as DNA synthesis and energy metabolism [153,154], while excess of iron can be lethal to bacterial cells. Under physiological conditions, iron mainly exists in two oxidation states, as oxidized Fe^3+^ (ferric iron) and as reduced Fe^2+^ (ferrous iron). Even though bacteria can absorb Fe^3+^ from the exterior, they quickly reduce it to the more soluble Fe^2+^. However, Fe^2+^ is an accelerator of ROS formation, resulting in a large amount of ^•^OH via Fenton and Haber-Weiss reaction (Fig. 4B) [134]. These radicals subsequently cause damage to the bacteria by lipid peroxidation in the cell membrane and by introducing detrimental protein and DNA modifications, leading to an accumulation of oxidative damage and subsequently to cell death.

Iron oxide nanoparticles (FeO-NPs) have a larger surface area than iron ions, and exhibit overall a higher antimicrobial activity. The major forms are magnetite (Fe_3_O_4_) and its oxidized forms, maghemite (γ-Fe_2_O_3_) and hematite (α-Fe_2_O_3_) [53]. First, FeO-NPs interacts with the bacterial cell by electrostatic interactions and subsequent cell adhesion to the cell envelope. Then, by penetrating the cell wall, they interact with lipids and proteins on the cell membrane, thereby changing the osmotic pressure and causing membrane disruption. Once inside the cell, FeO-NPs may trigger ROS generation and oxidative stress [135], thereby disrupting DNA replication and inducing DNA double-strand breaks [155].

### 7.1 Generation of reactive oxide species

Several studies report a higher sensitivity of Gram-positive bacteria to FeO-NPs than Gram-negative bacteria [18,155]. As outlined previously, the outer membrane of Gram-negative bacteria has an increased negative net charge due to its composition and the embedded LPS. Hence, the penetration of free radicals and lipophilic solutes are limited for which Gram-negative bacteria are thought to be less sensitive to FeO-NPs. Gholami et al. [11] evaluated the antibacterial activity of magnetic FeO-NPs (mFeO-NPs), ferrous and ferric ions against *S. aureus* and *E. coli*. In their studies under aerobic conditions, ferrous ions showed the strongest inhibitory effect against the growth of both stains. For ferric ions, the antimicrobial effect depended on its conversion to the ferrous form by the bacteria and the formation of the hydroxyl free radicals. This conversion is critical for the antibacterial activity of Fe^3+^. Intriguingly, mFeO-NPs showed the lowest antibacterial activity under aerobic conditions, probably because its activity relies on the release of ions. However, under anaerobic conditions, mFeO-NPs exhibited the strongest antibacterial activity, as mFeO-NPs need less oxygen than Fe^3+^ ions to produce ROS. Thus, NPs are capable of generating higher ROS levels in the absence of oxygen. Additionally, these studies suggest that not only the iron content and ROS production is crucial for the antibacterial activity of mFeO-NPs, but that many other factors may be involved. For example, the authors proposed that bacterial cell death could be actually triggered solely by the physical contact and interaction between the bacterial cell wall and the positively charged NPs.

To further examine interaction of the NPs with the bacterial envelope, and to address the impact of the surface potential of FeO-NPs, Arakha et al. [135] explored the antibacterial properties of both negatively charged FeO-NPs (nFeO-NPS) and positively charged chitosan coated FeO-NPs (pFeO-NPs) against *B. subtilis* and *E. coli*. The experiments showed a higher antimicrobial effect of pFeO-NPs than nFeO-NPs, probably due to electrostatic repulsion of the latter, resulting in a reduced or even abolished attachment of the nFeO-NPs. Nevertheless, exceeding a critical concentration of NPs in the culture media, antimicrobial activity was imposed also in the case of negatively charged nFeO-NPs, probably by NP-mediated ROS production in the exterior. In the case of the positively charged pFeO-NPs, a more localized ROS production is proposed due to the attachment of the nanoparticles to the bacterial surface, and a thereby more efficient reduction of bacterial cell viability (Fig. 4Ci). In both scenarios, the extracellularly produced ROS successfully depolarized the bacterial membrane, thus causing membrane damage, as evidenced by a LIVE/DEAD BacLight Bacterial Viability assay (Fig. 4Cii). Finally, the authors also studied the membrane depolarization of *B. subtilis* by SEM and the interaction of pFeO-NPs and the bacterial surface by EDX. Thereby, the authors could demonstrate that the interaction interface between the nanoparticles and the bacterial cell has an important role in the antibacterial effect of the iron oxide nanoparticles, explaining the higher activity of pFeO-NPs than nFeO-NPs. Thus, cell membrane disruption by direct interaction probably plays a greater role than metal-mediated ROS generation in case of the FeO-NPs.

In bacteria, iron represents an essential cofactor for many enzymes. But at elevated levels, iron can produce ROS by Fenton reaction. The generated radicals can subsequently damage the various biomolecules of the cell, such as lipids, proteins, and DNA. Importantly, both Gram-positive and Gram-negative bacteria can take up also the less-soluble Fe^3+^ and reduce it to the preferred Fe^2+^. However, the reduced form can generate large amounts of ^•^OH by Fenton and Harber-Weiss reaction (Fig. 4B). Iron-mediated ROS production can take place ubiquitously; *i.e*. in the cell environment, localized at the bacterial envelope and inside the bacterial cell. Excessive levels of extracellular ROS can lead to membrane lipid peroxidation and oxidation of membrane and membrane-associated proteins, leading to membrane rupture and leakage of intracellular components. Additionally, large amounts of intracellular ROS can break the bacterial antioxidant defense system, resulting in oxidation of cytosolic proteins and enzymes, DNA breakage and lipid peroxidation of the cell membrane [49,50].

The activity of FeO-NPs (magnetite nanoparticles) has been extensively explored over the last decades [11,154,156]. For example, Bukhari et al. [157] explored the anti-biofilm properties of FeO-NPs in root canal treatment based on its peroxidase-like activity, which allows the nanoparticles to catalyze H_2_O_2_ and thus generate free radicals. The authors suggested that the mechanism is based on the binding of H_2_O_2_ onto the iron oxide of the nanostructure with the subsequent activation of the H_2_O_2_ by the ferric or ferrous ions, producing ^•^OH, O_2_‾^•^ and HO_2_^•^. Their results showed that FeO-NPs in combination with H_2_O_2_ exhibited improved antibacterial properties during root canal treatment compared to H_2_O_2_ or FeO-NPs alone.

Later, Pallela et al. [158] synthesized hematite nanoparticles (αFeO-NPs) in *Slida cordifolia* extracts and determined its antimicrobial properties against *B. subtilis*, *S. aureus*, *E. coli* and *K. pneumoniae*. The authors examined two mechanisms for αFeO-NPs activity against Gram-positive and -negative bacteria. In a biological environment, the antimicrobial contribution by metal ion release was dominant. However, under UV condition and visible light, ROS generation is triggered by various mechanism, e.g. by introducing defect sites in α-Fe_2_O_3_, or by the generation of electron-hole pairs. The thereby generated free radicals, such as O_2_‾^•^ and ^•^OH, can subsequently disrupt the cell envelope by electrostatic, dipole-dipole, hydrogen bond, hydrophobic and van der Waals forces. This leads to disorganization and disruption of the cell envelope, and thereby to bacterial death.

## 8 Antibacterial activity of gold

Gold atoms exhibit a low reactivity, in particular regarding oxidation by dissolved oxygen. Thus, less free ions are released from Au ions and Au nanoparticles (Au-NPs), and less ROS are generated. Consequently, ROS generation and ion release are supposed to play a subordinate role in the antibacterial activity of gold and Au-NPs, while direct interaction with the cell envelope, and binding to intracellular components of the bacteria are thought to represent the key mechanisms.

For example, Cui et al. [159] noted that Au-NPs did not involve the ROS generation for killing bacterial cells, but described several targets of Au-NPs within the bacteria's metabolism and RNA transcription. Moreover, Zhang et al. [160] showed that to obtain an equivalent antibacterial effect as obtained by other metal nanoparticles, much higher levels of Au-NPs had to be used; e.g. 197 μg mL^−1^ Au-NPs equaled the effect of 4.86 μg mL^−1^ of Ag-NPs against *S. aureus*. Several studies on Au-NPs support the notion that the antimicrobial effect of Au-NPs is relatively weak or undetectable, while others report various levels of bacterial killing. Dasari et al. [161] demonstrated that both Au and Au^3+^ possess antimicrobial activity against *E. coli*, *Salmonella typhimurium* and *S. aureus* with only a slight difference among species. Additionally, they demonstrated a strong impact by the buffer, exposure time, NP concentration, the bacterial species and strain, highlighting the complexity of metal-mediated antimicrobial activity. Furthermore, an important but ill-defined parameter of many studies is the amount of residual ions in the nanoparticles, leading to discrepancies which further complicate the comparison of results [[162], [163], [164]].

Many studies have been performed using so-called Au nanoclusters (Au–NCs) as their surface to volume ratio is higher than in Au-NPs, increasing the potential interaction interface between the gold particle and the biological target system. For example, Zheng et al. [165] compared the antibacterial activity of Au–NCs and Au-NPs, and demonstrated that the nanoclusters killed 96% of *S. aureus* and *E. coli* cells, whilst the nanoparticles had a nearly 50-fold lower efficiency and reduced viability only by 3 and 2% in *S. aureus* and *E. coli*, respectively. Intriguingly, the authors identified ROS production as one of the antibacterial mechanisms of Au–NCs, probably due to its suggested enzyme-like catalytic activity of AuNCs.

### 8.1 Cell membrane disruption

The main mechanism of bacterial toxicity by Au-NPs is supposed to be linked to the direct adherence of the nanoparticles onto the cell surface via electrostatic interactions. Ortiz-Benitez and co-authors [32] found that Au-NPs can bind the lipids of the bacterial membrane by hydrophobic behavior and decompress the lipid bilayer. Their Au-NPs were absorbed on the lipid surface by coulomb forces and due to the dipole-charge, the phosphocholine changed from an inclined position to a vertical position. Subsequently, the strong electrostatic forces lead to the compaction of lipid molecules around the Au-NPs, resulting in a big and beyond-repair pore in the membrane leading to cell death. However, the authors specified that this mechanism may be specific for *S. pneumoniae*, as the same experiment performed in *S. aureus* and *E. coli* lead to increased cellular ROS production [165] and vesicle lysis [166], respectively.

### 8.2 Generation of reactive oxide species

Oxidative damage upon gold exposure was determined under aerobic conditions by Muñoz-Villagrán and co-workers [167]. The authors evaluated the generated ROS levels by using the ROS scavengers 2,2′-Bypyridyl (BPL) and ascorbic acid. The results showed that after co-treatment of cells with Au^3+^ and BPL (or ascorbic acid), the growth of *E. coli* improved due to the reduced levels of hydroxyl radicals. In addition, ROS generation was assessed by the fluorescent probes H_2_DCFDA and DHE, which detect ROS and superoxide, respectively. Treated cells depicted an increase in fluorescence after Au^3+^ treatment, indicating the production of ROS. Moreover, the DHE experiment demonstrated that the O_2_¯ molecule is the main ROS generated by Au^3+^ in *E. coli*. Using microarray gene expression analysis, Zheng et al. [165] demonstrated that Au–NCs created a strong metabolic imbalance that induces an upregulation of oxidative enzymes and a downregulation of reductive enzymes, thereby leading to an accumulation of intracellular ROS. These results further support the concept that internalization of Au–NCs can lead to increased ROS levels and oxidative damage.

## 9 Ion based biomaterials with antibacterial capacity

**Fig. 5:**
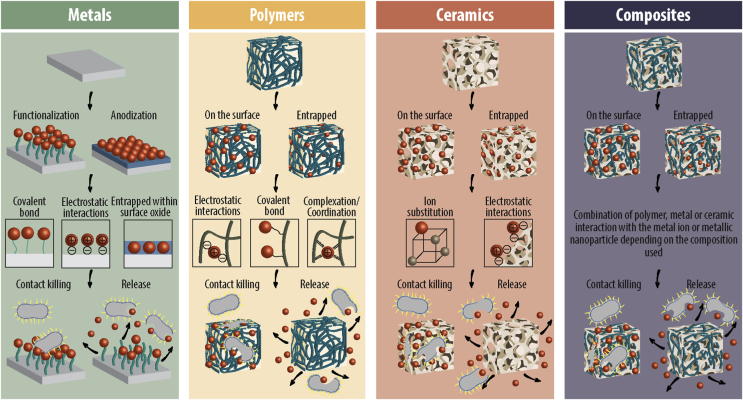
Schematic overview of different biomaterials modified with metal ions and/or metallic nanoparticles (red dots represent metal ions or metallic nanoparticles). Metal ions and nanoparticles can be entrapped in or coated on materials such as polymers, ceramics, metals, and composites, where they are hold in place e.g. by electrostatic interactions or covalent bonding, and thereby augment the material with antibacterial activity.

When metal-based nanomaterials are used without any support material, they often come with certain limitations, such as aggregation due to their high surface reactivity and a thereby reduced antimicrobial efficiency [168]. Hence, over the last years, many researches have focused on the immobilization or integration of M-NPs and M^n +^ onto biomaterials to enhance their antimicrobial properties. To do so, different immobilization routes have been developed: (1) incorporation and entrapment of M-NPs and M^n+^ in a porous matrix; (2) *in situ* generation of the M-NPs in the matrix support; (3) immobilization of the M-NPs or M^n +^ onto a functionalized solid support (Fig. 5). The choice of method depends on different factors such as size, morphology, type of functionalization, solid support to use, stability of the M-NPs, and the application for which the biomaterial is intended to be used [168].

Importantly, the surface chemistry and/or the chemical composition of the bulk biomaterial can be performed by both physical and chemical modifications. The probably most simple physical method is based on dipping the respective biomaterial into a solution of the antibacterial compound [169,170]. However, due to the lack of strong interaction between the biomaterial and the antibacterial agent, the loaded compounds typically show a fast and uncontrolled release. Thus, hydrogels and ceramics with a large pore size are becoming more and more popular in recent research, as these systems show well defined morphological structures and can be altered for the respective application. One key method to do so is the layer-by-layer self-assembly technique (LbL), in which the biomaterial is crafted by applying alternating layers of opposite charges by dip-coating or spray-coating [171,172]. In this process, M-NPs or M^n +^ can be embedded in a polymeric matrix such as cellulose or silk, resulting in a homogeneous dispersion throughout the biomaterial without aggregation due to the electrostatic interactions between the metallic agent and the molecules of the matrix [171,173,174].

Several chemical modifications of titanium or hydroxyapatite coatings have been developed by using anodic oxidation to add the M-NPS or ions. Depending on the engineered release rate, the bactericidal activity of the biomaterial can be modulated to the respective application. Furthermore, M-NPs and M^n +^ can be covalently bound to various polymers by the grafting method where reactive groups such as thiol groups or amine-terminated silane monolayers react with the metallic specimen [175]. Alternatively, the interaction process can be established by complexation or covalent conjugation between the M^n +^ or M-NPs and the reactive group [171]. Other metal-augmented bioactive materials are based on the incorporation of M^n+^ and M-NPs into ceramic materials by the ion substitution methodology and either total or partial metal ion replacement [176].

Here we will focus on the use of metal ions and nanoparticles in bone infection treatment. The biomaterials typically used can be divided into: (i) metals, (ii) polymers, (iii) ceramics and (iv) composites. Ideally these biomaterials should not only avoid bacterial adhesion and reduce microbial viability, but they also encourage the fast adhesion of the host cells and support tissue integration.

### 9.1 Metals-based biomaterials

Metals and its alloys have been used as biomaterials for a long time due to their excellent mechanical properties, machinability and biocompatibility. However, once they are in the body, they represent hot spots for bacterial infection. Especially silver ions and nanoparticles have been used in the direct coating of metal implants through various strategies such as electrodeposition. For example, silver ions have been deposited onto the surface of titanium samples, testing its antibacterial activity with mono-species biofilms (*Streptococcus sanguinis* and *Lactobacillus salivarius)* and with a multi-species oral biofilm model [177,178]. The results showed a significant decrease in viable bacteria on the treated samples. However, antimicrobial effectiveness was higher against single-species biofilms. The same authors [179], subsequently, tested the effect of the treatment onto dental implants in an *in vivo* study in beagle dogs during the first stage of peri-implantitis. The results showed a significant reduction of the infection around the dental implant, and therefore, a decrease in the bone resorption.

**Fig. 6:**
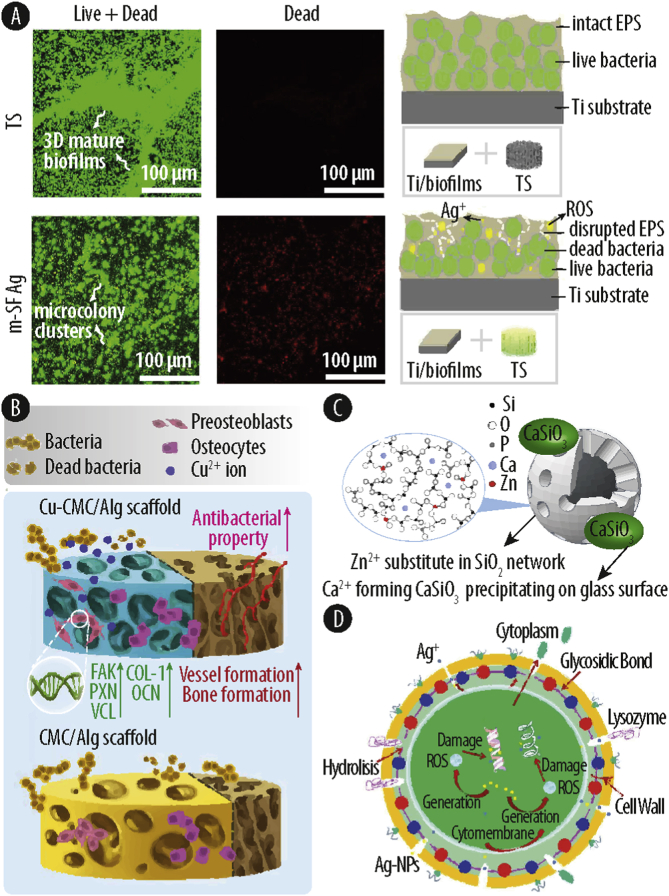
(A) Confocal laser scanning microcopy (CLSM) of bacterial biofilms treated with manufactured titanium scaffolds (TS) and after functionalization with nanosilver encapsulated silk fibrin (m-SFAg). The overlap of green and red signal yields green yellow. On the right, a schematic of the experimental design and results is depicted. Topical reactive oxygen species (ROS) and silver ions (Ag^+^) species are released from m-SFAg scaffolds and diffuse into vicinities of the biofilms and further degrade the biofilm EPS, thus exposing the embedded biofilm bacteria and inactivating them. **(B)** Schematic illustration of the antibacterial and osteogenic processes of Copper (Cu)-modified carboxymethyl chitosan (CMC) and alginate (Alg) (Cu-CMC/Alg) and (CMC/Alg) scaffolds *in vivo*. When the Cu^2+^ ions released from the Cu nanoparticles gradually cross-linked the polymer mixtures, which was further turned into a Cu-CMC/Alg scaffold with an interconnected porous structure by freeze-drying. The *in vivo* study demonstrated that the Cu-CMC/Alg scaffolds induced the formation of vascularized new bone tissue and avoided the clinical bacterial infection. **(C)** Schematic of the role of zinc (Zn) and calcium (Ca) in 80S (80SiO_2_–15CaO–5P_2_O_5_ in mol%) glass. Because of the Zn^2+^ substituted in the glass, it release was limited and no anti-methicillin-resistant staphylococcus aureus was detected. **(D)** Schematic illustrator of synergistic antibacterial mechanism of Lysozyme/Chitosan/Silver/Hydroxyapatite hybrid coating on Ti. In the early stage, lysosome hydrolyze the β-1,4-glycosidic bond of peptidoglycan on the cell wall of bacteria, resulting in the rupture of cell wall and the cytoplasm as well as the spilling of other intracellular substances. At the same time, chitosan (CS) can adsorb the negatively charged protein of cell wall, blocking the cell wall pore channels and leading to bacteria apoptosis with no nutrition exchange. The Ag^+^ released from Ag-NPs can penetrate into the bacterial cell and destroy or damage DNA by generation of intracellular ROS or direct contact. Reprinted with the permission from ACS Publications [173,174] and Elseiver [180,181].

Jia et al. [173] developed a fully porous titanium scaffold by metallic powder 3D printing, and subjected it to *in situ* hydrothermal growth of a micro/nanostructured titanate layer with nanosilver encapsulated in crosslinked silk fibrin (Fig. 6A). The silver-entrapped scaffold was then tested against *S. aureus*, showing a decrease in bacterial adhesion and an active killing for up to 6 weeks. The authors suggested that the antimicrobial properties were attributed to the high cargo loading (0.91% w/w), the durable topical Ag^+^ release (initial burst of 9.9% of the total nanosilver content, followed by a steady release, and 40.6% still remaining in the scaffold after 42 days of incubation), and the metal-induced ROS generation.

Likewise, titanium dental implants were functionalized with copper (Cu–Ti) by an electrochemical approach [182]. The authors observed that the amount of copper deposited on the surface depended on deposition time; showing that 5 min were required to cover 3–4% of the surface area. After culturing the Cu–Ti dental implants with *Porphyromonas gingivalis* (*P. gingivalis*), microbial viability was greatly reduced in the surrounding environment. The authors concluded that the copper released from the surface generated an antimicrobial “safe zone” (~30% of the deposited copper (17.7 μg per disc) was released within 14 days), thus improving implant healing. Tavakoli et al. [183] improved the antibacterial properties and corrosion of steel 316L using a PDMS-SiO_2_ and CuO-NPs coating. Due to the incorporation of CuO-NPs onto the metallic substrate, small irregular-shaped particles (3.5 ± 1.5 μm) and large agglomerations appeared, consisting of CuO and SiO_2,_ respectively. The antibacterial effectiveness was enhanced compared to PDMS-SiO_2_ coating, explained by the diffusion of the CuO-NPs into the bacterial solution and thereby killing the bacteria. Importantly, based on the analysis of SEM micrographs, the applied CuO nanoparticles got more agglomerated with increasing amounts, leading to a lower diffusivity and thus, to a lower antibacterial activity. However, this effect was only observed when the concentration of CuO-NPs was higher than 0.5 wt%. Another metal frequently used for its incorporation into metal-based implants is zinc. In particular, Huo et al. [184] developed zinc-based titania nanotubes (NT-Zn) where first the NT are fabricated by a two electrode configuration, followed by the incorporation of Zn by a hydrothermal treatment, and finalized by an annealing step at 450 °C. NT-Zn exhibited long-term capability to inhibit bacterial colonization with a gradual decrease of 20% over the first 7 days, and a diminished Zn burst due to the annealing treatment. This is the ideal scenario in clinical practice as it allows to control the desirable responses while avoiding potential side effects associated with Zn overdose.

To improve the biological performance of titanium substrates, a bioactive multi-layered structure was built via a LbL [171]. Zinc acetate (ZnAc_2_) solutions at different concentrations (5, 10, 20 and 40 mg mL^−1^) were used to dope a chitosan/gelation complex. Their results showed that the optimal modified Ti substrate was Ti-LBL-Zn10, having the greatest potential for promoting osteoblast growth while inhibiting bacterial adhesion and growth.

### 9.2 Polymers-based biomaterials

Another promising approach to fight infection is the combination of NPs and metallic ions into polymers, forming new biomaterials for different applications [169,170]. In the simplest design, NPs or metallic ions are absorbed to the polymer and coat the surface. In other situations, polymers can be grafted to or from a nanoparticle surface. To do so, the polymer chain needs to possess a reactive group (e.g. thiol group) that can covalently bind to a complementary NPs surface. On the other hand, polymers can be grafted from a NP surface by starting its polymerization from a molecule located on the particle surface. Each technique has its advantages and disadvantages and the method of choice depends on the desired application.

For example, Zakia et al. [175] developed a Ag-NPs modified alginate with improved antimicrobial properties. For its preparation, the authors photo-crosslinked the hydrogel by dissolving the methacrylated alginate, the Ag-NP solution and Irgacure. The Ag-NPs were homogenously dispersed without aggregations in the modified alginate due to the electrostatic interactions between the NPs and the alginate molecules. The authors also showed that the antibacterial activity of the hydrogel derived from the release of Ag ions out of the hydrogel and did not originate from the Ag-NPs themselves. Importantly, the release rate of the metallic ion in polymer gels can be optimized by the adjustment of the chemical nature as well as the physical structure of the gel matrix. Therefore, the release of Ag^+^ cations can be engineered for effective antimicrobial activity with prolonged efficacy.

The impregnation of alginate nanofibers with Ag-NPs was studied in the same context. Mokhena et al. [185] studied the impregnation of Ag-NPs into electrospun alginate nanofibers through their complexation with chitosan (CS). Ag-NPs were homogenously dispersed onto the surface and the release of the NPs was controlled by the chitosan coating. The results showed that growth of both Gram-positive and negative bacteria was inhibited for at least 24h. The authors attributed this to the release of the silver ions into the solution due to the porous structure of the nanofiber.

In addition to silver ions or nanoparticles, further studies deal with other elements immobilized in polymeric scaffolds. Regiel-Futyra and co-workers [186] developed a chitosan based films with Au-NPs. The modified chitosan films at 5 mM and 10 mM of Au-NPs created pores in the bacterial cell wall and induced structural changes in the bacterial membrane of *S. aureus* and *P. aeruginosa* as was observed by TEM. The Au-NPs released from the chitosan-based film decreased in a concentration-depend manner (1, 2, 5 and 10 mM were tested) the number of bacterial colonies by at least 10-fold *in vitro*. The authors suggested the use of these biomaterials as adhesive bandages for wound dressing or as antibacterial coatings.

Kao et al. [172] studied the antibacterial properties of polydopamine-coated titanium after spraying various metal ions (Cu^2+^, strontium (Sr^2+^) or Zn^2+^) onto the surface. The study found that although the effective concentrations of Cu (0.2, 0.5 and 1 wt%), Sr (2, 5 and 10 wt%) and Zn (2, 5 and 10 wt%) ions were higher than of Ag (0.02 wt%), their use can significantly decrease bacterial viability. Moreover, the bactericidal effect was correlated with the ion concentration gradient, confirming dose-dependency. Regarding the type of metal ion, they did not observe significant difference between Sr and Zn, however copper showed higher antibacterial efficacy than Sr and Zn.

Lu et al. [174] developed a Cu-modified carboxymethyl chitosan (CMC) and alginate (Alg) scaffold (Cu-CMC/Alg). (Fig. 6B). Instead of adding the copper ions to the polymer mixture and provoke an uncontrolled polymer cross-linking, the authors added Cu-NPs to the mix. Therefore, the release of copper ions from the Cu-NPs caused a gradually cross-linking of the polymer solution that later became Cu-CMC/Alg. Moreover, the presence of copper into the scaffold enhanced osteogenesis and promoted bactericidal properties. The results showed that Cu^2+^ ions were released slowly for the first 5 h. During the next 7 h, the released amounts significantly increased (approx. 3.1 μmol L^−1^ h^−1^), followed by a decrease over the next 12h (approx. 1.1 μmol L^−1^ h^−1^).

One compelling aspect of NPs is the possibility to functionalize them and to encapsulate other biologically active molecules that can synergize in the antibacterial effect or provide additional functionality. For instance, silver carbene complexes (SCC) were assembled into NPs [187]. These SCCs complexes showed low cytotoxicity and allowed for a variety of formulations for delivery and release. Another example are AgNPs decorated with graphene quantum dots (Ag-GQDs) coated with PEGylation and produced by a laser synthesis process [188]. These systems dramatically reduced the silver concentration required to inhibit bacterial growth due to the synergistic effect between Ag and GQDs. Additionally, the PEG coating enhanced the uptake of Ag-GQDs into bacterial cells.

In general, Au is required at high concentrations for antimicrobial killing. Thus, many efforts have focused on the use of Au-NPs. Due to its properties, Au-NPs can be easily functionalized with additional ligands that can, for instance, specifically interact with receptors located on the surface of the target cells. For example, Casciaro and co-workers [189] conjugated covalently the antimicrobial peptide Esc(1–21) to Au-NPs via a polyethylene glycol linker. This system was effective for the topical treatment of epithelial infections, at a very low concentration of NPs (5 nM), and showed an improved resistance to proteolytic digestion and an increased capability of decomposing the bacterial cell membrane.

### 9.3 Ceramics-based biomaterials

Innovative biomaterials that provide tissue regeneration and inhibition of pathogenic microorganisms are based on the incorporation and coating of M^n+^ and NPs onto ceramic materials. An important feature of hydroxyapatite is its ion substitution capability, that may occur with a total or partial metal ion [176]. This can be harnessed to M^n +^ -substitutes that provide apatite cements with antibacterial properties. Moreover, M^n +^ may be incorporated during the synthesis of bioactive glass nanoparticles (BGN) or shortly after their formation. Zheng et al. [190] developed an Ag-modified BGN by a post-modification method which results in the incorporation of silver by soaking the nanoparticles in silver nitrate. After several wash steps, the modified-BGN were calcined to stabilize the silver augmentation. The composition of the Ag-modified BGN corresponded to approx. 87SiO_2_-10.4CaO-2.6Ag_2_O (mol%) – as determined by EDS. The authors also studied the concentration of released Ag^+^ from the modified BGN, being 672 ± 113 μg L^−1^ after 24 h. Zheng and co-authors also described the presence of Ag-NPs in the BGN particles due to the high-temperature treatment which resulted in the reduction of silver nitrate into Ag-NPs. This probably allows for a sustained antibacterial activity, as Ag^+^ can be released in relatively short time to kill bacteria, while the Ag-NPs are expected to maintain their state for longer and thus provide a long-term activity.

The incorporation of silver into hydroxyapatite/polylactic acid (HA/PLA) coatings significantly contributed to enhancing the antibiotic properties of stainless-steel substrates. Yuan and co-workers [191] used the chemical precipitation method to synthesize Ag-doped HA powder to coat stainless steels samples by a spin-coating technique with Ag-doped HA/PLA. Notably, silver ions replaced calcium ions in the HA crystal lattice. The antibacterial activity of Ag-doped HA/PLA was tested against *E. coli* considering different [Ag]/[Ca] ratios and showed a 85% of bactericidal efficiency after 24h.

Zhang et al. [192] developed a silver-graphene oxide (Ag-GO) modified-β-tricalcium phosphate (β-TCP) scaffold by a combination of a three dimensional (3D) printing and LbL coating technique. The antibacterial activity of the scaffold was tested against *E. coli* and it was shown not only to effectively kill bacteria, but also to present positive effects on osteogenesis. Likely, Chen et al. [180] synthesized two 80S structure based materials (80SiO_2_–15CaO–5P_2_O_5_ in mol%): (1) Zn^2+^ substituted in the 80S structure (xZnO/80S) and (2) ZnO added in the 80S (80S + xZnO), where the ZnO coexists with the 80S (Fig. 6C). Then, it was possible to compare the antibacterial efficiency of the Zn^2+^ when it is incorporated to the bioglass by an ion-subsittution or onto the surface. The results showed that while incorporated Zn^2+^ (xZnO/80S) did not inhibit bacterial growth, the 80S + ZnO showed great antibacterial promise against methicillin-resistant *Staphylococcus aureus* (MRSA). This may highlight the importance of Zn^2+^ accessibility and release for ROS production and antimicrobial activity.

### 9.4 Composite-based biomaterials

Antimicrobial bioactive properties have also been achieved in scaffolds prepared from CS, HA and silver nanowires (Ag-NWs) [193]. For the later, a fast initial release of silver ions was reported, accompanied by a slow sustained release over time due to the reaction of the metallic silver of the Ag-NWs with O_2_. Moreover, the combination of CS and Ag-NWs resulted in a synergistic effect, with chitosan increasing the permeability of the cell wall and thus accelerating silver ions into the cell. The antimicrobial studies showed that the scaffold inhibited bacterial growth in both the cell medium and on the composite surface.

An hybrid coating based on lysozyme, CS, Ag-NPs and HA supplied metallic implants with highly effective antibacterial capability for a long time, and favoured the adhesion and proliferation of osteoblasts [181]. The given antibacterial mechanism is shown in Fig. 6D. In the earlier stage, lysozyme provoked the rupture of the bacterial wall. At the same time, the amino group of CS absorbed the negatively charged proteins of the cell wall, resulting in the blockage of the proteins and channels, leading to cell death. The Ag-NPs were used as a reservoir of Ag^+^ and thus sustained the antibacterial efficacy over time. Released Ag^+^ crossed the weakened cell wall, leading to intracellular ROS production, thus damaging various proteins and inhibiting bacteria proliferation.

Additionally, ZnO-NPs have been used to develop nanocomposites with carboxylated graphene oxide sheets (ZnO/GO-COOH) [194]. Due to the interaction between the negatively charged carboxyl groups on GO sheets and the positively charged Zn^2+^, the release of zinc ions is almost constant over two days, while for ZnO-NPs there is a strong Zn^2+^ burst at the first day. Moreover, only 11% of the total Zn^2+^ was released from the ZnO/GO-COOH after 12 days, while 47.7% were found to be released from the ZnO-NPs. These graphene-based composites have shown good biocompatibility and both osteogenic and antibacterial activity, highlighting their potential for bone substitution materials and bone regeneration.

### 9.5 Synergistic effect in biomaterials

As distinct M-NPs and M^n +^ employ different mechanism to kill bacteria, the combination of different metallic nanoparticles and/or ions can increase the antibacterial effect and simultaneously reduce cytotoxicity.

A recent study reported the co-implantation of Zn and Ag into titanium surfaces by plasma immersion ion implantation [195]. The modified titanium surfaces showed excellent osteogenic activity and antibacterial ability *in vitro,* which was attributed to the synergistic effect of the long- and short-range activity rendered by Zn and Ag ions, respectively. Another study [196], based on the development of bimetallic gold–silver nanoparticles (Au/Ag-NPs), was found to have great antimicrobial activity in a concentration-dependent manner, being more effective in inhibiting bacterial biofilm formation at 10 μM. The small and spherical Au/Ag-NPs penetrated through the tested biofilms and inhibited the entire bacterial populations achieving complete disintegration of the *E. coli* biofilm after 2h.

Matsuda et al. [197] studied the bacteriostatic and bactericidal properties of fluoride-containing ZnO–CuO (ZCF) nanocomposites. They inhibited the bacterial growth of *S. mutans* and showed great potential for its use in dental implants. Importantly, the results showed a stronger antibacterial effect for ZCF compared with the control lacking the fluoride (ZC), even though ZCF had a lower concentration of copper. Therefore, the authors concluded that different metals play complementary roles in the antibacterial mechanism. Hence, it is crucial to determine the correlation between metals and biological effects such as antimicrobial effectiveness or enzyme inhibition.

Recently, the generation of bioactive glasses (BG) doped with therapeutic and antibacterial ions have gained considerable interest. In this context, Bejarano et al. [198] incorporated Cu and Zn-doped BG (CuZnBG) into a poly (D,l-lactide) (PDLLA) based scaffold and studied its antimicrobial properties against MRSA. The release after immersion in cell culture medium showed a steady release of Cu^2+^ over time, while Zn^2+^ reached a maximum after day 1 and sharply decreased thereafter. Moreover, the total release of Cu^2+^ was at least 30 times higher than Zn^2+^. The distinct profiles probably originate from their differences in atomic radii and electronegativity, which impact the interaction with the non-bridging oxygen of the silica tetrahedrons from the glass network. The weaker binding of Cu to the glass structure may explain its higher release compared to Zn ions.

Silver and copper have been also added together with Ca(OH)_2_ onto titanium by electrochemically assisted deposition [199]. The addition of copper and silver ions led to a higher reduction of bacterial growth (25% bacterial activity) compared to the pristine Ca(OH)_2_ (40% bacterial activity). The results also showed a strongly different release profile for the two ions. While silver ions were constantly released over 17 days, copper release was only noticeable for the first 6 h. This was attributed to the lower total copper concentration in the titanium coating.

Additionally, a bimetallic Ag–Au-NP composite in a cellulose support matrix was developed by Hu and co-workers [200]. With a loading of 10 μg mL^−1^ of NPs, the nanocomposite showed notable antibacterial properties against both *S. aureus* and *E. coli*. The authors proposed that induced ROS generation by the catalytic action of the Au-NPs and the Ag^+^ release represent the main antibacterial mechanism of these nanocomposites.

Taken together, these studies document the high potential of combinatorial approaches to improve antimicrobial efficiency. The synergy of distinct metal ions and metallic nanoparticles can harness orthogonal routes of action, and e.g. combine the effect of physical membrane damage by the nanoparticle, followed by ion release and uptake into the bacterial cell, and thus metal-dependent ROS generation, and specific targeting of biomolecules. Moreover, due to the cooperative enhancement of antibacterial activity, the number of required ions and M-NPs can be reduced, thus minimizing the chance of detrimental effects in the host.

### 9.6 Preclinical and clinical studies in ion based biomaterials

Extending the understanding of the physical and chemical properties of metal ions and nanoparticles to *in vivo* situations will be key for their use as new biomaterials. Starting for example with mouse models, this could lead to predictive models for assessing their antibacterial properties before starting clinical trials. In that direction, Wang et al. [201] demonstrated that AgNPs coated on poly(gamma-glutamic acid)(ϒ-PGA) hydrogels promote wound healing in male BALB/c mouse; for example, after 14 days of treatment, the deposition of collagen and an intact epidermis layer was observed by histological analysis. Similarly, Heo el al. [202]. enhanced bone tissue regeneration by the use of gelatin-based hydrogels augmented with Au-NPs (Gel-Au-NPs), which were formed by UV irradiation of methacrylated gelatine (GelMA) and Au-NPs. The authors then checked the bone regeneration capacity in an *in vivo* model where defined defects were created in a rabbit bone and subsequently filled with distinct types of hydrogels: Gel alone (control), Gel-BMP (bone morphogenic protein), and Gel supplemented with Au-NPs at three different amounts (1, 5 and 14 μg of Au in 70 μL mQ). All the experimental groups showed a positive effect on bone healing compared to the control, but the highest increase in regenerated bone volume (RBV) was found in hydrogel supplemented with BMP and the highest amount of Au-NPs.

In 2017, Metin-Gürsoy and co-workers [203] reported a nanosilver coating on standard orthodontic brackets. When placed onto the mandibular incisors of Wistar Albino rats, the presence of *S. mutans* was significantly decreased compared to untreated brackets, and lead to a reduction of caries on the smooth surfaces. They also checked for silver cytotoxicity; no signs of argyria (i.e. a permanent blue grey discoloration of skin, nails, and mucous membranes) were observed, and that the serum concentration was found as 0.00175 μg L^−1^. Thus, the silver levels remained well below the thresholds allowed in clinical trials [204,205]. Xie et al. [206] developed a titanium implant coating consisting of HA, Ag-NPs and chitosan (HA/Ag/CS). By using polydopamine, they succeeded to avoid the fast release of silver ions from the coating, and the authors could demonstrated a 91.7%, 89.5% and 92% efficiency against *S. aureus*, *S. epidermidis* and *E. coli*, respectively. Moreover, after implantation of the coated titanium into rats, they found new bone formation in their longitudinal study.

Freire et al. [207] evaluated the antibacterial properties of a formulation termed Nano Silver Fluoride (NSF), which is composed of Ag-NPs, chitosan and fluoride. They could show that the NSF was effective against *S. mutans* growth in children's dental enamel biofilms; both CFU counts and absorbance values reflecting viability were reduced. Later, Tirupathi et al. [208] evaluated the clinical cariostatic efficacy of nano-silver incorporated sodium fluoride (NSSF) dental varnish compared to silver diamine fluoride (SDF) in inhibiting the progression of dental caries in primary molars. The study showed a better or equal efficacy of NSSF compart to SDF. But more importantly, NSSF did not provoke dark staining of dentinal tissue, and thus its use may be favoured.

Xie et al. [209] demonstrated the success of using quaternary ammonium (QA) capped with Au–NCs (QA-AuNCs) for combating multidrug-resistant bacteria in an *in vivo* mouse skin infection model. The authors successfully demonstrated its applicability against bacterial infections using a low dose of Au–NCs (<40 mg mL^−1^). Moreover, QA-AuNCs did not cause any detectable inflammatory response, and did not provoke drug resistance or toxicity in mammalian cells or animals. Additionally, Xu et al. [210] studied the synergistic antibacterial effect of Ag-NP coated titanium implants in combination with various antibiotics (vancomycin, rifampin, gentamicin and levofloxacin) in a longitudinal infection model in rats. The study showed a sustaining Ag release from the Ti implants for the first two weeks, thereby enhancing the bactericidal capability of the applied antibiotics.

In respect to orthopedic prosthesis, three metal-based antibacterial coating systems are currently the main proponents on the market. One of them is the endoprosthesis system MUTARS® (Implantcast, Germany), which consists of titanium-vanadium covered by a 15 μm layer of silver, using a gold layer of 0.2 mm thickness as a bonding layer. Alternatively, METS® prosthesis marketed as Agluna® (Accentus Medical Ltd, UK) contain ionic silver as a surface modification, which is adsorbed from an aqueous silver solution onto the implant after anodization of the titanium alloy. Finally, also Megasystem C® (Waldemar Link GmbH & Co. KG, Germany) has silver coated prosthesis in their implant portfolio. These PorAg® prosthesis possess a deep 1 mm thick silver-containing layer, and an external 0.1 mm layer of TiAg_20_N. Due to the interaction between these two layers, a controlled electron cloud is generated on the surface which can target the bacterial cell wall. This is in strong contrast to the uncontrolled release of metal ions typically associated with pure silver coatings. Thus several clinical studies could demonstrate that there were no local or systemic side effects of silver in patients with PorAg® implants [211]. However, none of these prosthesis have applied the silver coatings on the articulating surface or on the prosthetic stem [211,212], leaving room for improvement.

## 10 Conclusions and future perspective

Due to the increasing immanence of antibiotic resistant and the lack of new antibiotics on the market, metal-based materials represent an important cornerstone in antibacterial therapy. At high concentrations, metals are toxic to both eukaryotic and prokaryotic cells but specific metals are poisonous at low doses only in bacteria, thus avoiding harmful effects in the host. Despite extensive research over the last decades, the exact antimicrobial mechanism remains unclear for most (if not all) metal-based compounds. However, many studies attribute their antibacterial properties to metal-induced generation of ROS, in combination with (local) membrane disruption and interaction with biomolecules such as DNA and proteins. It is of outmost importance to comprehensively describe the underlying mechanism and to identify the crucial targets in bacterial cells, to be able to develop improved strategies and application-specific formulations.

With this review we aimed to highlight the most recent advances in antimicrobial research using Ag, Zn, Cu, Fe and Au ions and nanomaterials. We tried to outline and discuss the various mechanism of action which are currently discussed in the field. Importantly, in the case of nanoparticles, the release of metal ions creates a dual-mode of action where both NPs and ions can independently cause antibacterial effects.

ROS generation may happen both inside and outside of the bacterial cell and while local disruptions of the bacterial envelope may facilitate uptake of NPs and ions, extensive membrane rupture will directly lead to cell death as the bacterium cannot compensate for the leaked cell content. However, only few studies have so far focused on the impact of metals and metal-based nanoparticles on gene expression, protein synthesis and cell metabolism. Several proteins have been already identified as direct targets of metal ions, but many more are expected due to the ubiquitous use of metals as co-factors in enzymes and structural proteins. Thus, the increasing utilization of omics-based techniques will be one of the key drivers in the field in the near future.

One of the key problems in the field is the lack of unified standards, which severely complicates the comparison of the many excellent studies. For instance, the use of different bacterial strains, time points and varying compositions and designs of the metal-based compounds render it close to impossible to delineate common features and to compare the individual antimicrobial effects. Therefore, a standardized method to detect and measure the complex series of antimicrobial mechanisms in a time-dependent manner would be highly beneficial and clarifying. Hence, comprehensive studies are scarce and it seems impossible to capture all key parameters. Similarly, different bacterial species and often even different strains exhibit varying sensitivity to metal-based materials, further complicating the comparison of the published results. And even though there is a general agreement in the field that Gram-nature and cell surface composition play a crucial role, no clear pattern of metal susceptibility has been established to date, and thus no predictive models exist. Finally, most studies focus on free-floating (planktonic) bacteria in their studies. However, it is well known that most microbial life actually happens in complex, multi-species biofilms, where different bacterial species are embedded in a protective, self-produced matrix of extracellular polymeric substances. Consequently, increasing the complexity of the study design.

Without doubt, additional research is required to delineate the individual antibacterial mechanism to allow a deeper understanding of the individual sensitivity of bacteria to the various metal ions, nanoparticles and composites. It is highly likely that the combination of different metals ions is beneficial as it may lead to cooperative effects regarding their antimicrobial activities. Additionally, by modifying the nanoparticle surface and its physicochemical properties, both interaction with the bacterial cell wall and ion release could be fine-tuned and adjusted to the respective application. This would not only result in more efficient bacterial killing, but would also allow to lower the dosage of the applied compounds to mitigate side effects.

## Author(s’) disclosure statement(s)

All authors declare no competing financial interests.

## Funding statement

M.G-G and U.E have received funding from the postdoctoral fellowship programme Beatriu de Pinós, funded by the Secretary of Universities and Research (Government of Catalonia) and by the Horizon 2020 programme of research and innovation of the European Union under the Marie Sklodowska-Curie grant agreement No 801370. R.A.P is supported by the Spanish Ministry by the Ramón y Cajal Program (RYC2018-025977-I). Additional financial support was provided by the 10.13039/501100002809Government of Catalonia (2017 SGR 708) and MINECO/FEDER project (RTI2018-096088-J-100).

## Declaration of competing interest

All authors declare no competing financial interests.
